# Advances in tissue engineering of peripheral nerve and tissue innervation – a systematic review

**DOI:** 10.1177/20417314251316918

**Published:** 2025-02-05

**Authors:** Jayson Sueters, Rowan van Heiningen, Ralph de Vries, Zeliha Guler, Judith Huirne, Theo Smit

**Affiliations:** 1 Department of Gynaecology, Amsterdam UMC – location VUmc, Amsterdam, The Netherlands; 2 Amsterdam Reproduction and Development Research Institute, Amsterdam UMC, Amsterdam, the Netherlands; 3 Angiogenesis Laboratory, Cancer Center Amsterdam, Department of Medical Oncology, Amsterdam UMC – location VUmc, Amsterdam, The Netherlands; 4 Medical Library, Vrije Universiteit, Amsterdam, The Netherlands; 5 Department of Obstetrics and Gynecology, Amsterdam UMC – location AMC, Amsterdam, The Netherlands; 6 Department of Medical Biology, Amsterdam UMC – location AMC, Amsterdam, The Netherlands

**Keywords:** Biomaterials, innervation, peripheral nerves, signaling factors, tissue engineering

## Abstract

Although various options are available to treat injured organs and peripheral nerves, none is without limitations. Auto- and allografts are the first choice of treatment, but tissue survival or functionality is not guaranteed due to often limited vascular and neural networks. In response, tissue-engineered solutions have been developed, yet clinical translations is rare. In this study, a systematic review was performed on tissue-engineered advancements for peripheral nerves and tissues, to aid future developments in bridging the gap toward the clinic by identifying high-potential solutions and unexplored areas. A systematic search was performed in PubMed, Embase, Web of Science, and Scopus until November 9, 2023. Search terms involved “tissue engineering,” “guided,” “tissue scaffold,” and “tissue graft,” together with “innervation” and “reinnervation.” Original in vivo or in vitro studies meeting the inclusion criteria (tissue-engineered peripheral nerve/innervation of tissue) and no exclusion criteria (no full text available; written in foreign language; nonoriginal article; tissue-engineering of central nervous system; publication before 2012; insufficient study quality or reproducibility) were assessed. A total of 68 out of 3626 original studies were included. Data extraction was based on disease model, cell origin and host species, biomaterial nature and composition, and external stimuli of biological, chemical or physical origin. Although tissue engineering is still in its infancy, explored innervation strategies of today were highlighted with respect to biomaterials, cell types, and external stimuli. The findings emphasize that natural biomaterials, pre-seeding with autologous cell sources, and solutions for reproductive organs are beneficial for future research. Natural biomaterials possess important cues required for cell-material interaction and closely resemble native tissue in terms of biomechanical, geometrical and chemical composition. Autologous cells induce biomaterial functionalization. As these solutions pose no risk of immunorejection and have demonstrated good outcomes, they are most likely to fulfill the clinical demands.

## Graphical abstract

**Graphical Abstract:**
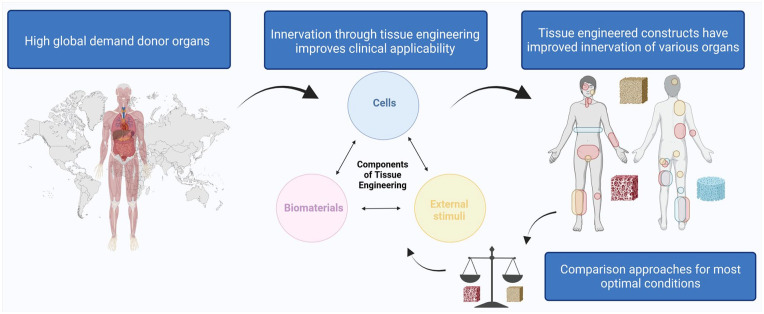

## Introduction

**Figure 1.**
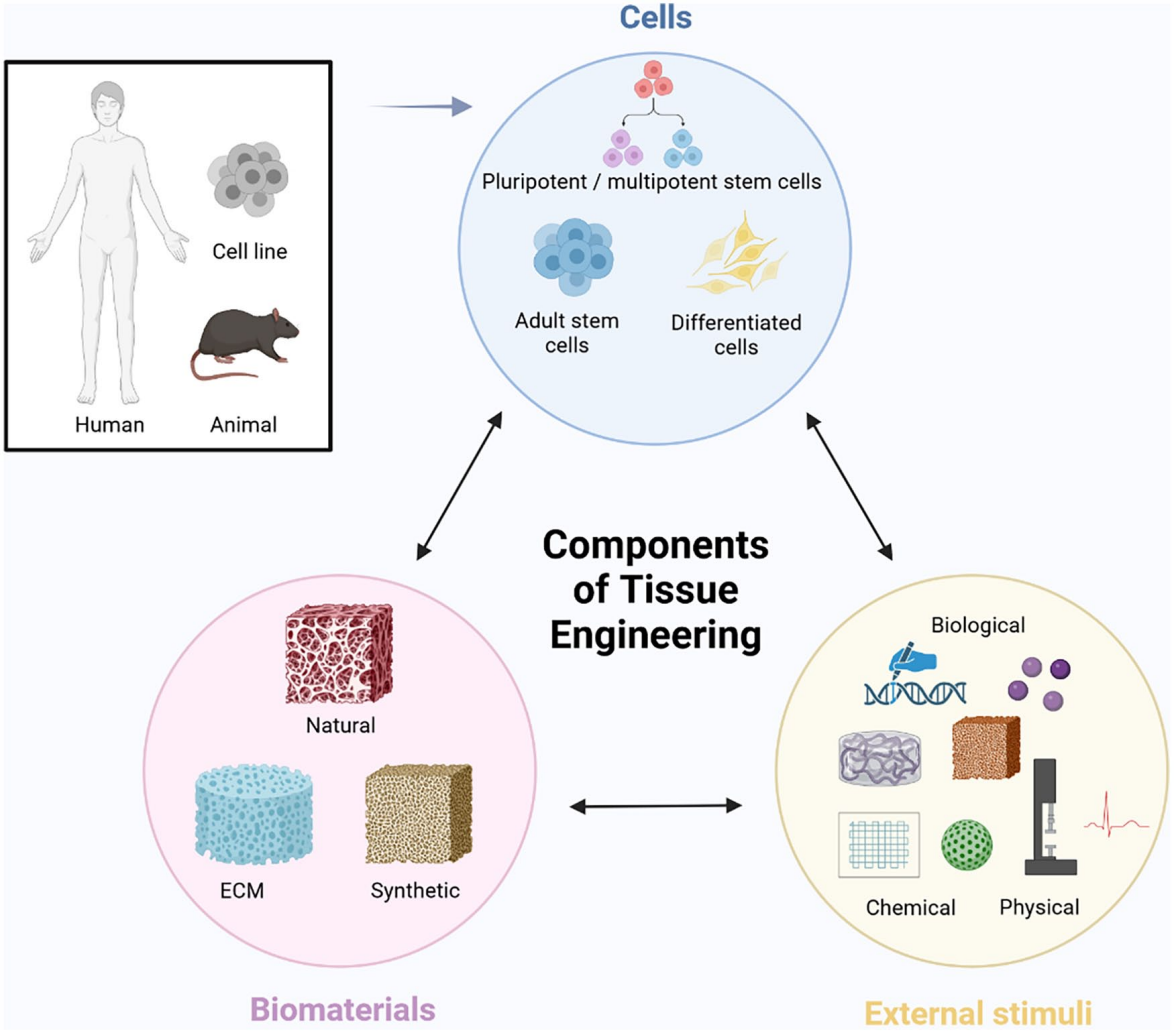
Modified tissue engineering triad consisting of: (1) cells, (2) biomaterials, and (3) external stimuli. (1) Cells are derived from human or animal tissue, or commercially available as immortalized cell line. They are classified as pluripotent/multipotent stem cells (=form numerous other cell types), adult stem cells (=form specific other cell types) and differentiated cells (=tissue specific mature cells, only form identical copies of themselves). (2) Biomaterials create a 3D micro-environment for cells and are classified as natural derivative (=produced by organisms), ECM derived (=from human or animal ECM) or synthetic components (=manmade materials). (3) External stimuli can be added to the engineered construct for further optimization, and are classified as biological (e.g. growth factors, bioactive molecules, gene modification), chemical (e.g. electrospinning, nerve leachate) or physical factors (e.g. mechanical stretching, plastic compression, physical therapy, electrical pulse).

Dysfunctional nerves, caused by physical trauma, disease, or congenital malformations, can substantially impact the quality of life.^1–6^ Especially peripheral nerve injuries are often encountered in clinical practice.^7^ Minor injuries may heal spontaneously, but more severe or extensive damage exceeds the body’s capacity to regenerate. Traditionally, treatment of damaged nerves or tissues involves transplantation of autografts, which is considered the gold standard.^8–10^ However, autografts are limited by their potential risk of donor site morbidity and incomplete functional recovery,^8,10–12^ and might not be sufficient to treat extensive injuries. Alternatively, allogenic grafts may be used but they require lifelong immunosuppressants to prevent rejection^13–15^ and often result in incomplete regeneration.^16^ Tissue engineering carries great potential to overcome these limitations. Tissue engineering (Figure 1) is based on a triad of cells, biomaterials, and external stimuli (e.g. growth factors, physical, and chemical stimuli),^17^ that are combined into an artificial construct to repair or replace tissues and organs. Not only the selected triad elements determine the potential for success, but also the element interactions are important and can drastically change by applications such as surface coating,^18–21^ three-dimensional (3D) structure modification^22–24^, and external stimuli.^25–27^ Due to the complexity of these interactions, finding the optimal combination is challenging.^28^

In the last decade, the number of publications on innervation of organs and peripheral nerve has increased, thereby suggesting a growing interest by clinicians and researchers in this field.^29^ In general, a two-dimensional (2D) in vitro assessment of biocompatibility, toxicity, degradability, and cell-material interactions is performed as first proof-of-principle.^29^ However, in vitro results may not translate well to the complex 3D micro-environment of the human body (e.g. synthetic meshes that cause inflammation,^30^ orthopedic implants unable to withstand stresses,^31^ or natural biomaterials lacking stability due to rapid biodegradation^32^). Therefore, in vivo follow up studies are conducted to provide insights into biocompatibility, toxicity, scar formation, and host responses (e.g. immune response, oxygen supply, and ECM formation).^29^ Finally, construct survival needs to be guaranteed for applications at clinically relevant sizes, which requires blood vessels for an adequate oxygen and nutrient supply.^33,34^ In addition, for the functional outcome of a transplant, innervation is vital.^35^ Typically, the recipient’s nerves gradually invade a transplant, a process called neoinnervation.^36^ This takes several weeks to months,^37^ thereby slowing down regeneration by delayed release of growth factors that are essential for cell growth, differentiation, and organization.^38^

Although tissue-engineering has been applied for many years,^17^ innervation is often overlooked. Most research is stranded before clinical application,^29^ which has so far limited the clinical use of tissue engineering to articular and elastic cartilage (nose, ear, and trachea), and treatment of skin and cardiovascular, mucosal and urological defects.^39^ In addition, large tissue-engineered constructs are often limited by insufficient formation of both a vascular and neural network, which are essential for tissue survival and functionality.^40^ Altogether, few tissue-engineered constructs really meet the demands of clinicians. An acceptable solution should guarantee biomechanical strength, preservation of geometry over time and temporarily matching the structural and mechanical properties of the target tissue, in absence of toxicity and immunorejection. Consequently, the use of tissue engineering for innervation in clinical setting is still relatively unexplored, and no commercial biomaterials are available today. Current solution are limited to the use of constructs for small nerve lesions (<3 cm)^41^ or innervation of small tissue volumes.^42^ Some FDA-approved, artificial nerve conduits have demonstrated satisfactional recovery but with significant side effects or regeneration failure.^41^ As innervation is essential for mobility and sensibility, this can cause issues with organ and tissue function, insensitivity, hypersensitivity, or chronic pain. Therefore, more research is needed for the development of new tissue-engineered solutions with better clinical outcomes.

Previously published reviews have extensively discussed specific aspects of tissue engineering for innervation, for example, specific biomaterial types,^29,43^ innervation of one body region,^44^ or state-of-the-art approaches.^45^ Despite the importance of the interplay between triad elements, to our knowledge, this has not been discussed and compared yet. Considering current limitations of tissue-engineered solutions for innervation, and the largely unknown knowledge of these element interactions, reviewing all tissue engineering elements combined will likely provide useful, new insights. Furthermore, the past has demonstrated us that medical developments may eventually find application in other organs or disorders than originally intended for. The most well-known example might be Viagra, which was originally developed as high bloodpressure medication, but is now widely used to stimulate erection. In terms of organs, porcine small intestinal submucosa (SIS) is a great example of a tissue-engineered alternative that was developed as artificial blood vessel,^52^ but in recent years has also found wide use in tissue reconstruction of bone, cartilage, bladder, and ureter.^46^ Therefore, the focus of this review was not restricted to a specific organ or tissue but included innervation of all organs and peripheral nerves, to identify common numerators and solutions that carry high potential to bridge the gap toward clinical application. Furthermore, by highlighting still unexplored areas, we hope to guide future research toward clinical outcomes.

### Motivation

Current treatment options for injury of organs and peripheral nerves are not ideal. These often lead to partial recovery, and unsatisfactory regeneration in case of severe damage. This gravely affects the patient’s quality of life. While autografts and allografts are often the first choice of treatment, both are associated with significant drawbacks and complications. Especially for grafts at clinically relevant sizes, the formation of a neural network is often insufficient and endangers the functional outcome. Recently, a growing number of tissue-engineered solutions have been developed to address these problems. Thereby offering new hope, but final clinical translation is generally not achieved. This review aims to identify high-potential biomaterials, cells, and external stimuli applied over the last 10 years for innervation of organs or periperhal nerves, and the challenges toward their clinical translation. Furthermore, we aim to bridge the gap between in vivo proof-of-principle and clinical application, by providing an overview of relatively unexplored areas to aid future developments.

### Method

PRISMA guidelines^47^ were followed after protocol registration and publication in PROSPERO (CRD42023402013) on February 5, 2023.

#### Eligibility criteria

Inclusion criteria: (i) an original study on in vivo implantation or in vitro organ systems for tissue-engineered peripheral nerve/innervation of tissue, (ii) written in English, and (iii) published after peer-review.

Exclusion criteria: (i) study on tissue-engineered central nervous system, (ii) published before 2012, of (iii) of insufficient quality or reproducibility.

A study was considered to be of insufficient quality if ethical approval or a defined disease model was absent. Furthermore, in vivo research had to have ⩾6 weeks follow-up and report all details on animal species, strain, sex, age, and number. In vitro studies required documentation of cell origin (cell line or species), isolation method, passage number, density/confluency, cell type, and experiment duration.

#### Search strategy

A strategic, bibliography search (Supplemental Table S1) was performed by a medical information specialist (R.d.V) in PubMed, EMBASE, Web of Science, and Scopus, from inception until November 9, 2023 (last updated). The search (with Medical Subject Headings and closely related terms) involved “tissue engineering,” “guided,” “tissue scaffold,” and “tissue graft,” along with “innervation” and “reinnervation.” The “snowballing” method was applied to references of all included articles and a Google Scholar search on an initial 200 hits was performed (J.S.) A database was created in Mendeley 1.19.4 (Mendeley Ltd.) after duplicate removal in Endnote X20.0.1 (Clarivate™).

#### Study selection

Two independent researchers (J.S., R.v.H.) screened all titles and abstracts for eligibility in Rayyan 1.2.1 (Rayyan Systems, Inc.),^48^ with blinding of authors and journals. The remaining studies were assessed for full content and exclusion had to be motivated by a criterium. Conflicts were resolved by a third author (J.H.). Articles were only included if originally written in English or that had a full English translation available.

#### Data extraction

Data extraction (J.S., R.v.H.) was performed using a predefined form, that included: author, publication year, strategy, biomaterials (type and composition), cell types (their origin and host species), external stimuli (of biological, chemical, or physical origin), and the disease model.

#### Quality and risk of bias assessment

A thorough quality assessment without methodological filtering^49,50^ was conducted independently by two researchers (J.S., R.v.H.) using the Joanna Briggs Institute Checklist for Quasi-Experimental Studies (JBI-Exp) and the Quality Assessment of Diagnostic Accuracy Studies-2 (QUADAS-2) tool for in vivo studies, and the Joanna Briggs Institute Checklist for Analytical Cross-sectional Studies (JBI-Ana) with the NIH Quality Assessment Tool for Observational Cohort and Cross-sectional Studies (NIH) for in vitro studies. In addition, the risk of bias was assessed using the Newcastle Ottawa Scale (NOS)^51^ for in vivo studies, and the Quality Assessment Tool For In Vitro Studies (QUIN).^52^

## Results

### Study characteristics

**Figure 2.**
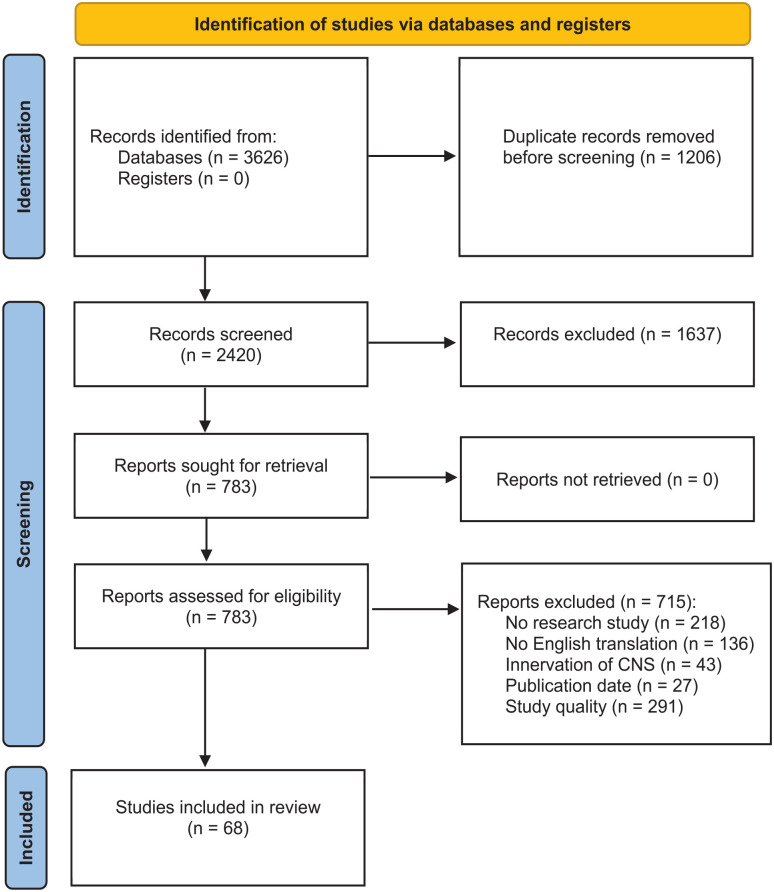
PRISMA 2020 flow diagram for the systematic review. During the identification phase, 3626 records were identified from the bibliographic databases PubMed, Embase.com, Web of Science and Scopus, with removal of 1206 duplicate records. The remaining 2420 records were screened and 1637 records were excluded based on title and abstract. All 783 reports were retrieved and assessed for eligibility. In total, 715 reports were excluded and 68 reports included in the review.

Our systematic search resulted in 3626 retrieved articles (Figure 2), that involved 1206 duplicates and 1637 articles that were excluded based on their title and abstract. In total, 783 studies were screened for full-text content and resulted in 68 eligible studies. Of the 715 excluded articles, 218 articles were not peer-reviewed (e.g. poster presentations, conference abstracts), 136 articles did not have an English translation available, 43 studies reported innervation of the central nerve system, 27 studies were published before 2012 and 291 studies were excluded because of insufficient quality.

**Figure 3.**
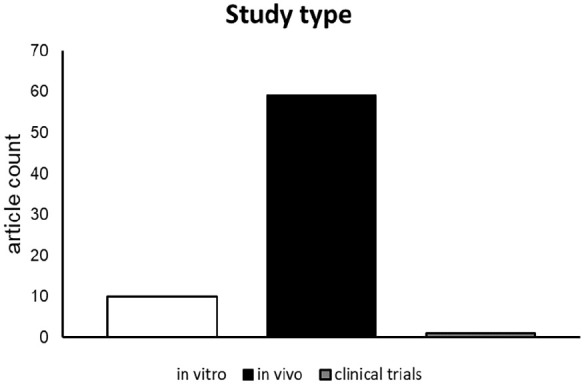
Quantity of reports on in vitro-studies, in vivo-studies and clinical trials.

**Figure 4.**
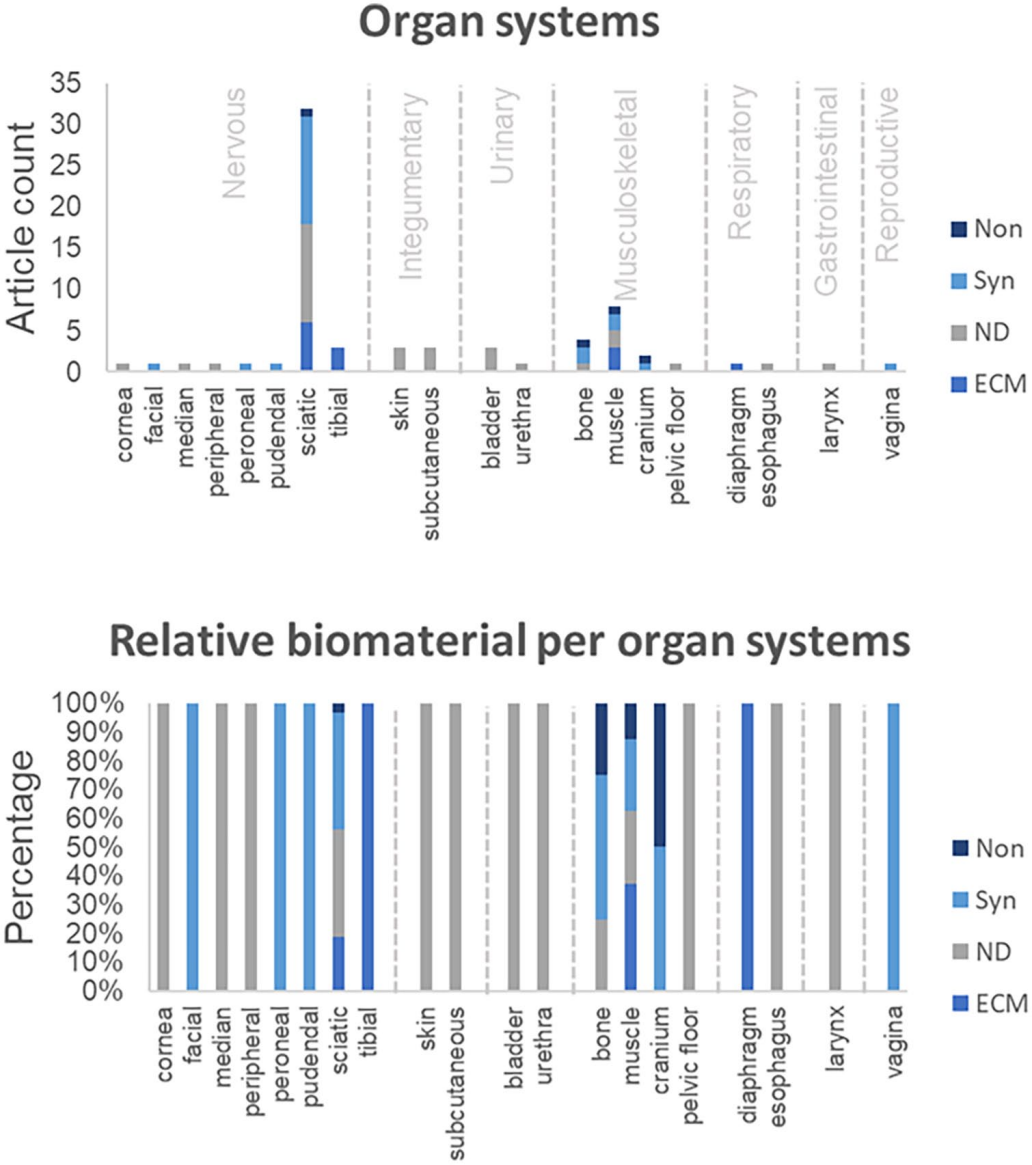
Reports with disease model on the nervous, integumentary, urinary, musculoskeletal, respiratory, gastrointestinal and reproductive organ system. Parts of the nervous system have been examined for cornea, facial, median, peripheral, peroneal, pudendal, sciatic and tibial nerves. Parts of integumentary system have been examined for skin and subcutaneous implants. Parts of urinary system have been examined for bladder and urethra. Parts of the musculoskeletal system have been examined for bone, muscle, cranium and pelvic floor muscle. Parts of respiratory system have been examined for diaphragm muscle and esophagus. Parts of the gastrointestinal system have been examined for the larynx. The reproductive system has been investigated for vagina. A) Quantity of included reports per organ system and based on the biomaterial origin classified as synthetic (Syn), natural (ND) or ECM-derived (ECM) material, or classified as cell-based research without use of a biomaterial (Non). B) Relative biomaterial origin utilized for examination of organ system sections.

**Figure 5.**
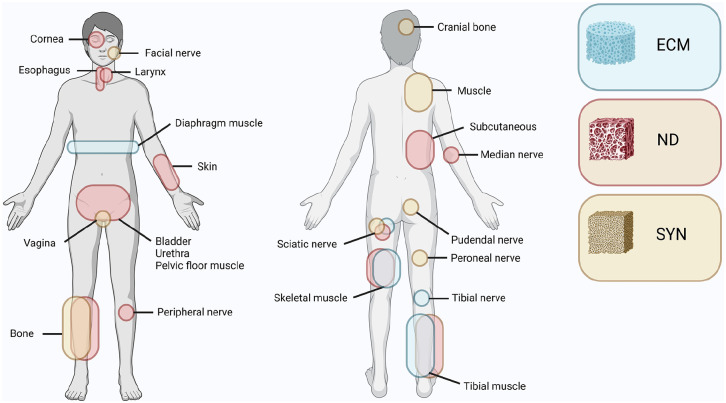
Schematic representation of tissue engineered organs based on the use of ECM-biomaterials (blue), naturalderived biomaterials (red) or synthetic materials (yellow).

Within the 68 included articles, a diverse selection of biomaterials, cell types, external factors, and target organs were reported. In total, the reports involved 31 natural-derived biomaterials (Table 1), 11 ECM-derived biomaterials (Table 2), 22 synthetic biomaterials (Table 3), and four scaffold-free approaches. Furthermore, the articles reported 57 in vivo animal studies, 10 in vitro studies,^18,24,25,53–59^ and only one clinical trial (Figure 3).^60^ Most animal studies involved rats and mice,^9–11,13–16,20–22,26,59,61–98^ but also rabbits,^99,100^ pigs,^101,102^ sheep,^103^ dogs,^104^ and Rhesus macaques were used.^105^ In total, peripheral nerves were reconstructed in 41 studies, while 27 studies focused on the musculoskeletal-, integumentary-, respiratory-, gastrointestinal-, urinary-, and reproductive systems (Figures 4 and 5). With respect to cell types, pluripotent stem cells were covered in one study, multipotent stem cells in 15 studies, adult stem cells in 5 studies, and mature cells in 10 studies. In terms of external stimuli, 20 studies reported biological-, 4 studies (bio)chemical- and 23 studies physical stimuli, whereas 28 studies combined stimuli.

**Table 1.** Studied natural-derived biomaterials with the applied disease model, host, properties, cell-seeding, external factors, and stimuli. Properties are indicated as positive (+), negative (−), positively altered (+/−) or unreported (?), with increase (↑), decrease (↓) or no changes (◯) in mixtures.

Biomaterial	Derived from	Applied in	Host	Compatible	Degradable	Innervate	Vascularize	Geometry	Modified biomechanics	Recover function	Cells	Factors	External stimuli
Chitosan^62,76,80,91^	Crustacean shells	Median nerve Sciatic nerve	Rat	+	+	−	+/−	+	+	+/−	SC	BDNF NGF LN	
Chitosan mixture (SF, COL, FIB)^20,53,54,70,98^		Sciatic nerve Skin	Rat	↑	↑	↑	↑	◯	↑	↑	BM-MNC BM-MSC DRG FB HUVEC HDMEC KC SC SN	BDNF GDNF NGF LN FN VEGF	Electrospinning
Hyaluronic acid mixture (hp, DA)^69,114^	Microbial fermentation	Cornea Subcutaneous	Mouse	+	+	+	+	−	+	+/−	AD-MSC CSK iPSC-NC UDSC	BDNF GDNF NGF IGF VEGF	3D printing
Alginate^18^	Seaweed	Skin		+	+	+/−	−	−	+	?	BM-MSC		
Agarose mixture (GEL)^73^	Red algae	Sciatic nerve	Rat	+	−	+/−	?	+/−	+	+/−	DRG MN	BDNF CNTF GDNF NGF CT-1 LN CH	
Collagen^59,64,74,83,92,95^	Animal tissue	Bladder bone Larynx Sciatic nerve Subcutaneous	Rat Mouse	+	+	+/−	+/−	+	+	+	AD-MSC BM-MSC HUVEC MPC SkMb SMC SVF	BDNF VEGF ACC	Gene modification Plastic compression
Fibrin(ogen) mixture (AGA, CH)^9,79^	Human plasma	Sciatic nerve	Rat	+	+	+/−	?	−	+	+/−	AD-MSC DRG SC		Electrospinning
Gelatin^55^	Denatured collagen	Skeletal muscle		+	+	+/−	?	−	+	+/−	iPSC PSM	BDNF CNTF GDNF	Micropattern
Gelatin mixture (FB, HA, AGA)^65,66,71^		Pelvic floor muscle Subcutaneous TA muscle	Rat Mouse	◯	↑	↑	↑	↑	↑	↑	DRG HCH HUVEC MPC	VEGF AGR	3D printing
Silk (fibroin)^25,75,99,101,102^	Arthropod production	Bladder Esophagus Peripheral nerve Urethra	Rat Rabbit Pig	+	+	+	+	+	+	+		LN	Micropattern electrical
Silk (fibroin) mixture (HA)^78^		Sciatic nerve	Rat	↑	↑	◯	◯	↑	↑	◯	DRG		

**Table 2.** Studied extracellular matrix-based biomaterials with the applied disease model, host, properties, cell-seeding, external factors, and stimuli. Properties are indicated as positive (+), negative (−), positively altered (+/−) or unreported (?), with increase (↑), decrease (↓) or no changes (◯) in mixtures.

Biomaterial	Derived from	Applied in	Host	Compatible	Degradable	Innervate	Vascularize	Geometry	Modified biomechanics	Recover function	Cells	Factors	External stimuli
dAM^61^	Human	TA muscle	Rat	+	+	+	+/−	+/−	−	+	AD-MSC		HIIT
ANG^13,14,63,68,72,97,104^	Human (sural) Rat (sciatic) Dog (sciatic)	Sciatic nerve Tibial nerve	Rat Mouse Dog	+	+	+	+/−	+	−	+	AD-MSC BM-MSC SC	GDNF Nerve leachate	Side-to-side bridge
BAM^26,196^	Porcine	skeletal muscle TA muscle	Human Mouse Rat	+	+	+	+/−	+/−	−	+/−	MPC		Mechanical stretch Physical therapy
DdECM^67^	Mouse	Diaphragm muscle	Mouse	+	+	+	+/−	+	−	+			

**Table 3.** Studied synthetic biomaterials with the applied disease model, host, properties, cell-seeding, external factors, and stimuli. Properties are indicated as positive (+), negative (−), positively altered (+/−) or unreported (?), with increase (↑), decrease (↓) or no changes (◯) in mixtures.

Biomaterial	Derived from	Applied in	Host	Compatible	Degradable	Innervate	Vascularize	Geometry	Modified biomechanics	Recover function	Cells	Factors	External stimuli
Poly-ε-caprolactone^10,22,82^,^84,86,90^	Polymerization ε-caprolactone	Pudendal nerve Sciatic nerve	Rat Mouse	+	+/−	+/−	?	+	+	+/−	BM-MSC DRG MPC SC	NGF LN PDL	Electrospinning 3D printing
Poly-ε-caprolactone mixture (dECM)^96^		Muscle	Rat	↑	◯	↑	↑	◯	◯	◯	AD-MSC MD-MSC		Electrospinning
Poly-L-lactide-co-ε-caprolactone mixture (COL, HA)^11^	Polymerization ε-caprolactone and L-lacide	Sciatic nerve	Rat	+	?	+/−	?	+	+	+/−	NCSC SC		
Poly ethylene dioxythiophene mixture (AGA)^87^	Polymerization 3,4-ethylene dioxythiophene	Peroneal nerve	Rat	+/−	−	+	?	+/−	+/−	+/−			Electrodeposition
Poly glycolic acid mixture (COL)^77^	Polymerization glycolide	Facial nerve	Rat	+	+	+/−	?	+	+/−	+/−	DFAT		
Polylactic-co-glycolic acid mixture (CH, PNS)^85,88^	Copolymerization PGA and polylactic acid	Sciatic nerve	Rat	−	+	+/−	+/−	+	+/−	+/−	SC		
Poly-L,D-lactic acid mixture (PGF)^16^	Polymerization L- and D-lactide	Sciatic nerve	Rat	+	−	+/−	?	+	+	+/−	DRG		
Polysulfone mixture (AGA)^15^	Polymerization oxy-1,4-phenylenesulfonyl-1,4-phenylene	Sciatic nerve	Rat	+/−	?	+/−	?	+	+	+/−		FKN	
Polypropylene mixture (UBM)^105^	Polymerization polypropylene	Vagina	Macaque	+/−	+/−	+/−	?	+	−	+/−			
Silicone mixture (COL, FIB, PA)^56,93^	Polymerization siloxane	Muscle sciatic nerve	Rat	+	−	+/−	?	+	+	+/−	AD-MSC CMC		Micropattern
ß-TCP Ceramics^24,100^	Synthesize tricalcium and phosphate	Bone	Rabbit	+	+	+	+	+	+	−	BM-MSC SC		3D printing
GelMA mixture (Li-Mg-Si)^94^		Cranial bone	Mouse	↑	◯	↑	↑	◯	↑	↑	HUVEC RUVEC		3D printing
Carbon nanotube yarn (SIL)^21^	Vapor deposition acetylene	Sciatic nerve	Rat	+	−	+	?	+	+	+			Electrospinning

### Biomaterials for innervation

**Figure 6.**
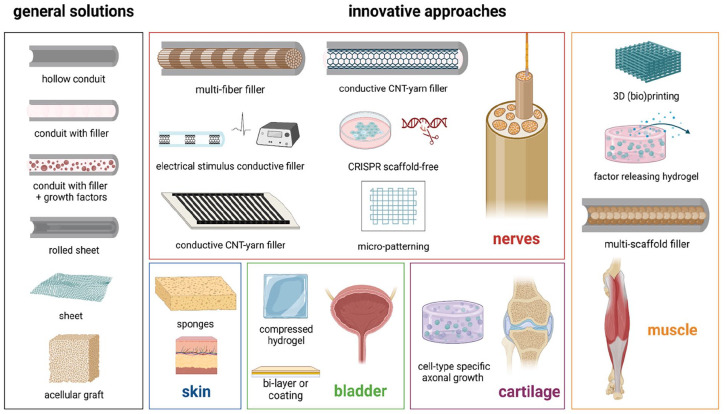
Schematic representation of general and innovative approaches with respect to the biomaterials applied for reconstruction of nerves (red), skin (blue), bladder (green), cartilage (purple) or muscle (orange).

In general, tissue engineering is based on biomaterials, that provide a native-like 2D or 3D micro-environment for cells. Biomaterials are either fabricated from nonabsorbable synthetic polymers, or are biodegradable and derived from natural- (e.g. chitosan, collagen, silk) or extracellular matrix-materials (e.g. bladder acellular matrix, acellular nerve graft).^106^ These types substantially differ in physical and chemical properties, and their interaction with cells is optimized through modification of for example, internal structure, chemical composition, and dimensions.^107^ For nerve regeneration, a biomaterial should be biocompatible, biodegradable without toxic by-products, have appropriate biomechanical properties (including mechanical stability) and geometrical dimensions that support nerve growth,^17,42,108^ and be electrically conductive to guide and extend invading neurons from the recipient.^16,42,108,109^ Furthermore, clinical applicability desires the material to be flexible, suturable, transparent, and resistant to collapse and tension.^28,29,110^ Within the 68 included articles, both a large number of general and innovative biomaterials (Figure 6) were applied.

#### Natural-derived biomaterials

Natural-derived biomaterials mainly consist of polysaccharides, proteins, or natural polyesters.^29,108^ These have been widely studied for innervation (Table 1) and, combined with cells or a host, 29 included studies reported the use of natural-derived biomaterials. This involved 23 in vivo- (rodents/rabbits) and 6 in vitro studies.

##### Polysaccharides

*Chitosan (CH)* is derived from chitin through deacetylation, and has found application as a conduit for sciatic-^20,62,70,79,80,91,98^ and median nerve,^76^ and as biomaterial for innervation of skin.^18,54^ Hollow CH conduits show excellent properties and can reduce inflammation,^85^ but they lack mechanical strength^108^ and cues to promote nerve regeneration.^20,70^ Therefore, they require addition of protein-based biomaterials,^20,62,70,79,98^ extracellular matrix molecules, or seeded cells. Adding silk fibroin or fibrin improves their degradation rates,^79,98^ and use of a peptide-mimicking filling can promote cell growth,^62^ nerve fiber growth, myelination, and fastens motor function recovery.^70^ Mechanical properties of CH are enhanced by acetylation, double-layering and biomaterial blending. Today, two-chambered CH conduits^76^ have been demonstrated to enhance functional regeneration of median nerve, and CH-collagen sponges have been applied for in vitro tissue-engineered skin^54^ and to assess the angiogenic effects of growth factor.^53^

*Hyaluronic Acid (HA)* is the largest glycosaminoglycan component of the human body and is important for various cellular processes.^69,111^ HA has excellent biomechanical properties for nerve regeneration except for its low mechanical strength.^29,112^ Therefore, it is usually cross-linked, or applied as injectable hydrogel.^113^ HA has demonstrated to improve vascularization, cell survival, myogenic differentiation, and innervation of the urethral sphincter,^69^ neuritogenesis of the cornea^114^ and functional recovery and accelerated innervation of pelvic floor^71^ and tibial muscle.^66^

*Alginate (ALG)* is highly flexible but requires chemical modification, physical crosslinking, or polymer blending to enhance its mechanical strength and to prevent inflammation.^29^ In vitro, alginate sheets have demonstrated to enhance neuronal growth.^18^

*Agarose (AGA)* has an ECM-mimicking structure but it needs functionalization and polymer blending for effective use.^115^ AGA has been shown to accelerate axonal regeneration, maturation, and innervation for muscle function recovery and nerve regeneration in rats.^9,73^

##### Proteins

*Collagen (COL)* is a major ECM component.^29^ It facilitates cell adhesion and movement,^116^ and can guide axonal regrowth.^117^ To improve their biomechanical stability, COL hydrogels often require compression, cross-linking, or polymer blending.^20,53,54,118^ COL has demonstrated effective for innervation of skeletal muscle,^95^ and regeneration of larynx^64^ and sciatic nerve.^74,83^ In compressed form, COL has been shown promising for the regeneration of cartilage and bladder wall.^64,92^ Furthermore, biomineralized COL can mimic bone in nanostructure, composition, and various biological functions, and it aids the formation of vasculature and neuronal networks.^59^

*Gelatin (GEL)* is formed from hydrolyzed COL and is extensively used for medical purposes.^29^ However, it often requires cross-linking, material blending, or pairing with enzymes to prevent collapse,^65^ or to create application-specific properties.^119^ GEL has demonstrated effective for functional recovery of nerves through controlled release of incorporated growth factors,^73^ and for promotion of muscle maturation and myoblast alignment.^55^ Furthermore, fibrinogen- or thrombin-paired GEL enables cell type-specific axonal growth by inhibition of migratory cells and differentiation of non-migratory chondrocytes.^65^

*Fibrin (FIB)* is a glycoprotein involved in blood clotting and is formed spontaneously from *Fibrinogen (FB)* and thrombin^65^ during nerve regeneration. It guides axonal regrowth, and Schwann cell migration and proliferation.^120^ Although FIB hydrogels lack mechanical strength, FIB fillers are suitable to promote fibrin alignment and nerve regeneration and function.^9,79^

Where *Silk (S)* is historically used as a suture material,^121^ *Silk Fibroin (SF)* is applied for repair of bone, ligament, cartilage, and skin because of its adjustable elasticity, flexibility, degradability, and resistance to fracture and compression.^29,122^ Furthermore, SF conduits and fillers have effectively promoted sciatic nerve regeneration in rat models.^78,98^ However, specific conditions apply as SF conduits filled with 200 S-fibers promote axonal regeneration with increased myelination, innervation and functional recovery, but fewer fibers provide insufficient nerve support, and more fibers physically hinder nerve growth.^78^ Bi-layered SF matrices are more suitable to aid recovery of the bladder, urethra, and anterior esophageal wall.^75,99,101,102^ However, formed rabbit urethra have shown to lack full regeneration of smooth muscle.^99^ In rat esophagus, SF matrices outperform small intestinal submucosa by presenting less shrinkage, inflammation and fibrosis.^75^ For porcine bladder, SF can improve the capacity and compliance,^102^ but acellular S presents more native-like structure and function, and regeneration of smooth muscle and urothelial tissue.^101^ Electrical stimulation of micropatterned or conductive-coated S can further enhance its effect on nerve alignment and nerve fiber growth.^25^

#### Extracellular matrix-derived biomaterials

Extracellular matrix (ECM)-derived biomaterials, from either animals or humans, undergo a process of physical or chemical decellularization.^123,124^ This removes all individual-specific, cellular components (e.g. deoxyribonucleic acid), and their associated risk of graft rejection by the recipient’s body.^28,125^ Acellular biomaterials posses an excellent biocompatibility,^126^ and are valuable because they contain preserved structural, mechanical, and biochemical cues that encourage nerve and tissue regeneration.^13,14,68,127^ Although mechanical properties of ECM-derived biomaterials are not easily tailored, 3D printing of bio-inks is possible.^128^ In this review, 12 studies reported ECM-derived scaffolds (Table 2) from the sciatic nerve, amniotic membrane, or bladder, that were tested in 10 rat/mouse-based studies,^13,14,26,61,63,67,68,72,97,129^ one dog-based study^104^ and one clinical trial.^60^ ECM biomaterials derived from humans were studied in rats, and the clinical study involved a porcine xenografts.

*Acellular Nerve Grafts (ANGs)* from human sural-^13,14^ and rat/dog sciatic nerve^63,68,72,97,104^ have shown effective in repair of long gap injury of sciatic^14,63,68,97,104^ or tibial nerve^13,72^ in rats. ANGs are also capable of species-dependent enhancement of myelinated axon density and functional recovery of plantar, tibialis anterior.^14^ For instance, peripheral nerve repair by human ANG is poor in rats,^13,14^ where euthymic rats show necrosis, lower innervated muscle weight and axon diameter, and fewer axons compared to athymic rats.^13^ Furthermore, neurite outgrowth of mice neurons with mice-derived laminin outperforms that when in contact with human-derived laminin.^14^ In addition, cell-seeding can enhance the ANG performance by guidance of neurite ingrowth through the release of bioactive molecules.^104^ Through seeding with motor and sensory-derived Schwann cells, isograft-like results have been achieved.^68^ In addition, ANG is enhanced by nerve leachate (for autograft-like function and increased nerve volume),^63^ thermal decellularization (for reduced immunogenicity and increased muscle action)^97^ and surgical attachment to a recipient’s nerve (for improved muscle recovery, senescence, and sprouting).^72^

*Denervated Tissues* are allogenic, acellular grafts^129^ that outperform synthetic materials in repairing tissue function, muscle volume, and innervation of diaphragmatic hernias in mice.^67^ A balance between ECM remodeling and skeletal muscle regeneration is therein key to success.^67^

*Bladder-derived acellular matrix (BAM)* can attract neurons and muscle fibers at implantation sites in mice,^60^ and is able to restore the function of tibialis anterior muscle in rats by enhancing vascularization.^26^ In a clinical study, BAM implantation resulted in angiogenesis within 5–8 weeks, and dense tissue with blood vessels, active skeletal muscle regeneration, and complete scaffold breakdown were seen within 6–8 months.^60^ However, two-fifths patients lacked a strength improvement, due to anterior compartment syndrome and the absence of a pre-operative measurable force.^60^

#### Synthetic biomaterials

Synthetic biomaterials, especially polyesters, are extensively used in tissue engineering because of their easy modification.^17,108,115,125^ In total, 22 included studies applied synthetic biomaterials (Table 3), that were either tested in small animals (19 studies) or in macaque primates (1 study).^105^

*Poly(ε-caprolactone; PCL)* is a hydrophobic polymer,^108^ that provides controlled protein release.^130^ Rolled PCL sheets have shown effective as nerve conduits to repair sciatic lesions in rats, by showing autograft-like axon regeneration, myelination, and innervation of muscles and skin, albeit with reduced wet muscle weight.^84^ PCL sheets are also suitable for treatment of stress urinary incontinence, as they induced sphincter contractions under electrical stimulation, and enhanced leak point pressure and muscle formation in rats.^86^ As nanofibers, PCL can improve axon numbers, neuromuscular innervation, and electro-physiological function of rat sciatic nerves,^90^ whereas melt-extracted PCL enhances functional motor recovery.^82^ Furthermore, aligned PCL fibers can support morphology, proliferation and trophic activity (Fibroblast Growth Factor) of mesenchymal stem cells, and adhesion of Schwann cells (SCs), for tissue replacement, innervation, and motor function recovery in rat sciatic nerves.^22^ Incorporation of Mg2+-releasing hydrogel in PCL biomaterials can promote neurite outgrowth, peripheral nerve regeneration, and functional recovery,^10^ but also optimizes biomechanical properties of dermal ECM for in vitro muscle regeneration.^96^

*Poly(L-lactide-co-ε-caprolactone; PLCL)* has excellent mechanical properties,^131^ and combined with collagen hydrogels it provides cell type-dependent support for sciatic nerve regeneration in rats.^11^ Furthermore, stem cell-seeded PLCL can improve electrophysiological function and gastrocnemius muscle recovery.^11^

*Poly(glycolic acid; PGA)* has been used as artificial nerve conduits, that significantly enhanced axonal outgrowth, maturation, and physiology of rat facial nerves.^77^

*Polylactic-co-glycolic acid (PLGA)* is hydrophobic and requires polymer blending or coating with ECM proteins to induce hydrophilicity for biomedical applications.^132^ Porous PLGA-polylactic acid conduits can enhance neurogenesis, axonal regeneration and myelination in rats.^88^ Although PLGA degradation products are acidic, they can be neutralized by CH degradation products to inductive a micro-environment for SC migration and guidance of axon regeneration in rat sciatic nerves.^85^ IT has further been demonstrated that one PLGA scaffold inside a conduit significantly enhances muscle functionality and M2 macrophage levels, while two or three PLGA scaffolds increase M1 macrophages.^85^

*Poly(L,D-lactic acid; PLDLA)* has been used at the interface of aligned phosphate glass microfibers (PGFs) and as conduit filler, and has shown increased axon regeneration and innervated muscle volume in rat sciatic nerves.^16^

*Polypropylene (PP)* is commonly applied as suture material. However, in treating pelvic organ prolapse (POP), its non-degradability adversely affects the structural and functional integrity of vaginal smooth muscle.^105^ PP combined with urinary bladder matrix has been shown to induce remodeling in rhesus primates, by increasing small smooth muscle bundles with normal contractility.^105^

*Silicone (SIL)* is widely used in biomedical applications as it is easily sterilized and has a high elasticity, thermal stability, and versatility in manufacturing.^133,134^ As conduit filled with COL, SIL is able to restore muscle function after denervation,^81^ and, combined with fibrin glue^93^ or micro-patterned polyacrylamide,^56^ it can restore sciatic nerve injury.

β-tricalcium phosphate (β-TCP) is a hard *ceramic* usually applied for bone regeneration.^24,31^ Both 3D-printed (P-β-TCP) and porous β-TCP (N-β-TCP) are able to promote Schwann cell (SC) growth, proliferation, and expression of growth factors (e.g. beta-nerve growth factor, neurotrophin-3, platelet-derived growth factor, and vascular endothelial growth factor), but a spindle-shaped SC morphology requires P-β-TCP.^24^ β-TCP scaffolds have shown effective enhancement of bone neural regeneration and osteogenesis through neurotization in rabbits.^100^ Furthermore, Li-Mg-Si ceramics combined with gelatin-methacryloyl (GelMA) have been reported to regenerate cranial bone defects in mice, but with a dependence on seeded human or rat umbilical vein endothelial cells.^94^

*Polyethylene dioxythiophene (PEDOT)* is non-degradable and the mechanism behind nerve regeneration by their electrical stimulation is unknown.^87^ PEDOT-AGA hydrogel has been found to support cell adhesion and neural regeneration of rat sciatic nerve injury.^87^

*Carbon Nanotubes (CNTs)* are non-degradable, electrically-conductive tubes.^21^ Conduits filled with 2% CNT yarn (cYarn) are able to improve walking and nerve function, and axon regeneration of rat sciatic nerves with a efficacy dependence on the concentration.^21^

*Polysulfone (PSF)* is a thermoplastic, conductive polymer,^135^ that can provide an anti-inflammatory, pro-neurogenic environment in rat sciatic nerve injuries.^15^ Fractalkine-treated compared to interleukine-4-treated PSF has demonstrated enhanced axon regeneration, regulatory macrophage levels, and migration of Schwann and endothelial cells.^15^

#### Scaffold-free approaches

While most tissue engineering approaches rely on a biomaterial for structural support, scaffold-free methods are being explored as well.^57,58,89,103^ Bone-ligament-bone scaffolds have shown successfull regeneration of sheep anterior cruciate ligament (ACL), with rapid host-cell migration, enhanced vascularization, innervation, and collagen density and alignment.^103^ More recently, also sheets from CRISPR-modified, adipose-derived stem cells have demonstrated functional recovery and myelination in rat sciatic nerve injury.^89^

### Cells for innervation

**Figure 7.**
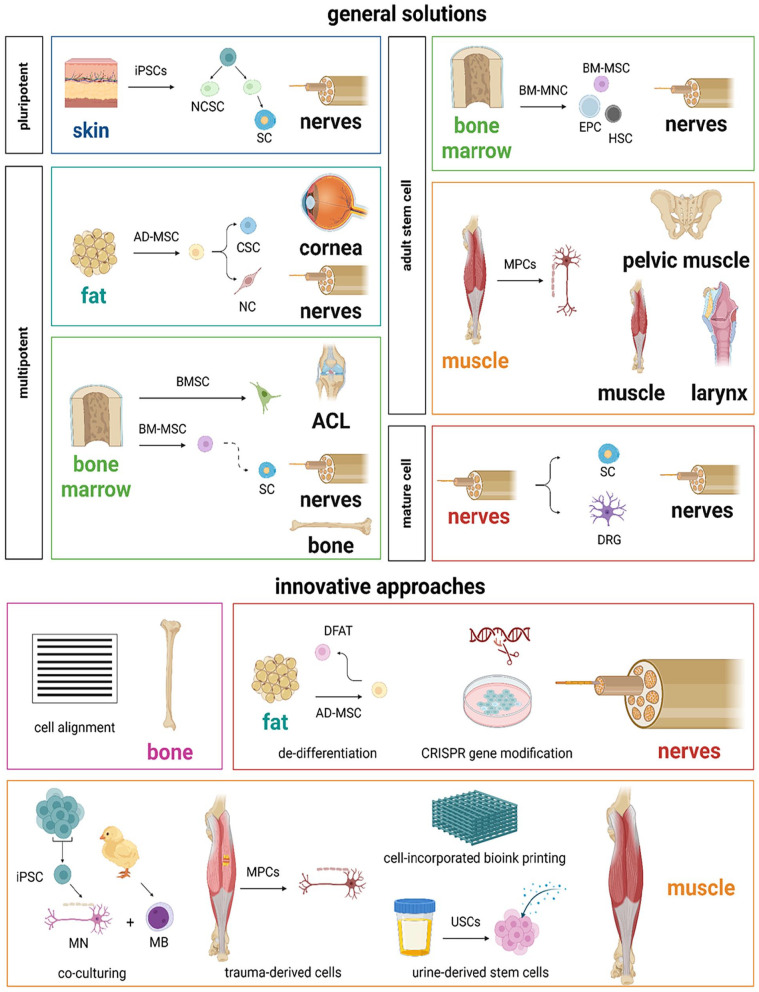
Schematic representation of general cell approaches with retrieval from nerves (red), skin (blue), fat (light blue), bone marrow (green) or muscle (orange). Innovative approaches have been applied to reconstruct bone (purple), nerves (red) or muscle (orange).

The human nervous system contains neurons and glia cells for transmission of nerve impulses.^136,137^ It further relies on cell-cell and cell-ECM interactions and signaling factors for communication, and for cellular guidance and fate.^137,138^ Glial cells provide structure, and attract and guide neurons through secretion of neurotrophic factors.^137^ Glial cells are either Schwann cells that aid conduction of nerve impulses,^139^ or Satellite cells that provide nutrients.^136^ Although implantation of a biomaterial can lead to host-driven innervation, this requires several months to complete.^37^ The addition of cells can accelerate this process and increase functionality. Within the 68 included articles, common and innovative cell approaches (Figure 7) were examined and 19 articles involved in vivo demonstrations (Table 4).

**Table 4.** In vivo studied cell types on biomaterial constructs with the origin specie, disease model, host, implantation type, compatibility, potency, biomaterial source, external factors, and stimuli. Cell types are classified as pluripotent stem cells, multipotent stem cells, adult stem cells, and mature cells. Potency and immune compatibility is indicated as high (++), good (+), compromised (−), of high concern (−−) or not applicable (◯).

Biomaterial	Derived from	Applied in	Host	Implantation	Immune compatible	Pluripotent	Multipotent	Biomaterial	Factors	External stimuli
iPSC^55^	Human	Skeletal muscle			◯	+	++	ND		
NCSC^11^	Human	Sciatic nerve	Rat	Xenogeneic		+	+	SYN		
AD-MSC^9,61,63,89,93,96^	Rat	Muscle Sciatic nerve TA muscle	Rat	Autologous Allogenic	++ −	−	+	ECM ND SYN	BDNF GDNF NGF Nerve leachate	Electrospinning HIIT
BM-MSC^20,22,59,97,100^	Human Rabbit Rat	Bone Sciatic nerve	Mouse Rabbit Rat	Allogenic Xenogeneic	−−	−	+	ECM ND SYN	BDNF LN FN PDL	Electrospinning
BMSC^103^	Sheep	Bone	Sheep	Allogenic	−	−	+			
MD-MSC^96^	Rat	Muscle	Rat	Allogenic	−	−	+	SYN		Electrospinning
UDSC^69^	Human	Subcutaneous	Mouse	Xenogeneic	−−	−	+	ND	NGF IGF VEGF	
DFAT^77^	Rat	Facial nerve	Rat	Syngeneic	++	−	+	SYN		
BM-MNC^98^	Rat	Sciatic nerve	Rat	Allogenic	−	−	−	ND		
MPC^26,66,71,86^	Human Rat	Pelvic floor muscle Pudendal nerve TA muscle	Rat	Allogenic Xenogeneic	−	−	−	ND SYN	AGR	Electrospinning Mechanical stretching 3D printing
DRG^10,73,79,82^	Mouse Rat	Sciatic nerve	Rat	Allogenic	−	−	−	ND SYN	NGF LN	3D printing
MN^73^	Rat	Sciatic nerve	Rat	Allogenic	−	−	−	ND	BDNF CNTF GDNF NGF CT-1 LN	
SC^11,20,62,68,70,79,85^	Human Rat	Bone Sciatic nerve	Rat	Allogenic Xenogeneic	−−	−	−	ECM ND SYN	BDNF LN FN	

#### Pluripotent stem cells

**Figure 8.**
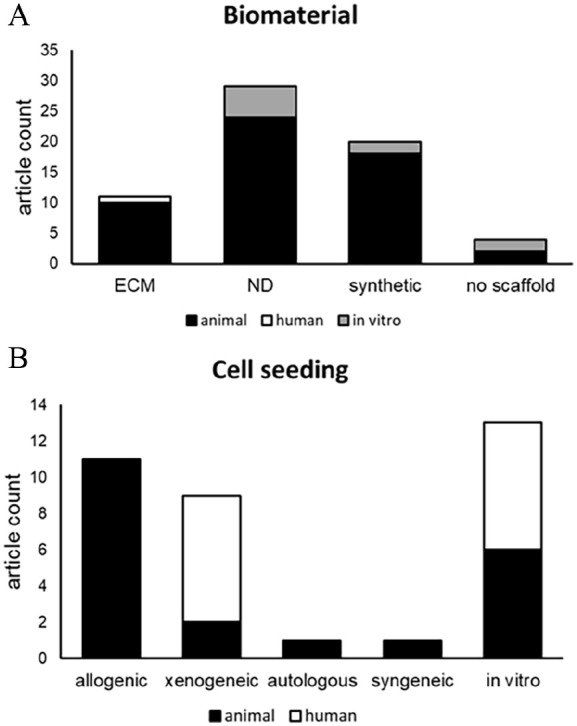
A) Quantity of reports on biomaterials derived from ECM-, natural- or synthetic-materials or without the use of a scaffold, for implantation in animals (blue), humans (orange) or in vitro examination (grey). B) Quantity of reports on seeded-cells of animal- (blue) or human-origin (orange) on biomaterials, for tissue engineered allogenic, xenogeneic, autologous or syngeneic implants or *in vitro* examination.

Stem cells poses a high capacity of self-renewal and differentiation, but they can also form malignant cells.^140^ Furthermore, autologous stem cells can enhance tissue regeneration and angiogenesis, and reduce inflammation without risk of an immune response.^141^ Most current stem cell-based research for innervation involve allogenic or non-human cells (Figure 8), as autologous cells were only studied once. Similarly, human cells have only been used to conduct xenogeneic implantation and to perform in vitro studies.

*Induced Pluripotent Stem Cell (iPSC)*-derived neural crest stem cells (NCSCs) and Schwann cells (NCSC-SCs) have both shown able to interact with new host axons and stimulate neurotrophic and nerve growth factors in repair of rat sciatic nerve.^11^ However, only NCSCs were found able to improve muscle recovery and axon distribution, and differentiated into SCs without teratoma formation.^11^ Furthermore, motor neurons from human iPSCs can form neuromuscular junctions with improved structure, function, and innervation, when combined with muscle constructs from especially chicks.^55^

#### Multipotent stem cells

Due to associated ethical concerns with embryonic stem cells, research on multipotent stem cells from adult tissue is more common.^42,140,142^ These cells can differentiate into bone, cartilage, and fat, to improve the local micro-environment and accelerate tissue regeneration and recovery.^143–147^

*Bone Marrow Mesenchymal Stem Cells (BM-MSCs)* from rabbits that were seeded on bone scaffolds have demonstrated to improve bone formation, blood flow, and nerve fiber growth.^100^ Micro-environmental modification by minerals, fibrillar hydrogels, and co-cultures can further enhance their impact on osteogenic gene expression, CD31+ cell numbers and vessel formation.^59^ Furthermore, their capacity to innervate nerves is likely associated with secretion of soluble factors^22,97^ and their ability to differentiate into Schwann-like cells.^97^ Moreover, BM-MSCs can be outperformed by human-traumatized muscle-derived MSCs or dental pulp stem cells under specific conditions.^57,58^

*Bone Marrow Stromal Cells (BMSCs)* originate from the same source as BM-MSCs and they deposit ECM protein to modify their environment. This might be more important than their presence as BMSC-seeded scaffolds in sheep with ACL injury have shown acellularization and sequential host cell repopulation, without improved regeneration or remodeling.^103^

*Adipose-Derived Mesenchymal Stem Cells (AD-MSCs)* can secrete neurotrophic factors with prolonged gene activation when paired with CRISPR, and thereby enhance functional recovery, nerve innervation, axon regeneration, and myelination.^89^ The performance of AD-MSCs varies with cell state and environmental factors. For instance, AD-MSCs can partially recover rat sciatic nerves,^9^ but neurally differentiated AD-MSCs demonstrate a greater functional effect.^93^ Corneal stromal cells from differentiated human AD-MSCs have shown to induce neuritogenesis with fast and enhanced in vitro maturation of neurite outgrowth in the cornea.^114^ Furthermore, (mixtures of) growth factors like nerve leachate can induce SC-like AD-MSCs with fast and improved nerve function.^63^ For regeneration of bladder wall, AD-MSC-derived smooth muscle cells (SMCs) compared to regular SMCs show enhanced secretion of angiogenic factors, tissue formation and innervation.^92^ However, AD-MSC-seeded muscle flaps were inferior in terms of torque and vessel formation.^96^ Also stromal vascular fraction cells are derived from adipose tissue and known to induce vascularization and innervation of muscle.^95^

*Urine-Derived Stem Cells (USCs)* are easily obtainable and can differentiate into endothelial, skeletal muscle, smooth muscle, and neurogenic cell lineages.^69^ USCs exposed to myogenic, angiogenic, and neurogenic growth factors, have demonstrated great muscle innervation in mice.^69^

*De-Differentiated Fat Cells (DFATs)* carry a multipotent capacity and have been shown to promote rat facial nerve regeneration with enhanced whisker motion, myelination, and fiber numbers.^77^

#### Adult stem cells

Adult stem cells are undifferentiated cells located in differentiated tissues.^140^ Although they are rare, low in potency and have an unknown lifespan,^148^ they are capable of specific differentiation and long-term repopulation.^149^

*Muscle Precursor Cells (MPCs)* from rats seeded on PCL sheets have been used to study the treatment of stress incontinence.^86^ Therein, MPCs were crucial for contractile activity and to restore normal leak point pressure without inflammation.^86^ Furthermore, MPCs from human gracilis muscle incorporated in bioink have demonstrated fast innervation and successful recovery of muscle volume and function for repair of pelvic floor muscle in immunocompromised rats.^71^ However, the immunogenic impact of xenogeneic MPCs has not been studied and is therefore unknown.^71^ Rat MPCs on BAM have demonstrated inconsistent functional recovery of volumetric muscle loss, as only 8/12 rats displayed improvement of torque with normal muscle fiber distribution after 6 months.^26^ MPCs co-cultured with AD-MSCs have shown enhanced vocal fold function in laryngeal nerve injury,^64^ and combined with neural crest cells they can improve functional muscle recovery in rats.^66^

*Bone Marrow Mononuclear Cells (BM-MNCs)* are a mixture of stem and progenitor cells. They are more readily available than BM-MSCs and have shown autograft-like regeneration of rat sciatic nerve injuries by demonstrating significantly increased myelination, nerve fiber density, and motor endplates in gastrocnemius muscle.^98^

#### Mature cells

As a direct innervation approach before transplantation, tissue-specific mature cells can be used instead of stem cells to form an interface for host neurons and fasten scaffold functionalization.^37^

*Schwann Cells (SCs)* are derived from embryonic, neural crest stem cells,^11^ and evolve into myelin- or non-myelinated types that are both found in the nervous system.^140^ SCs aid in debris clearance and inflammation control during nerve repair,^62,139^ but they also promote axon growth and myelination by secreting neurotrophic factors.^62^ Innervation by SCs was examined in five in vitro^24,62,70,79,85^ and two in vivo rat studies.^20,68^ SCs showed improved regeneration of rat sciatic nerve compared to BM-MSCs,^20^ and they demonstrated parallel alignment on printed β-TCP scaffolds in absence of signaling cues from neighboring cells.^24^ Furthermore, transplantation of ANGs that were seeded with allogenic rat sensory- or motor-derived SCs both demonstrated enhanced nerve regeneration and function.^68^ Moreover, in vitro neurite growth of SCs can be enhanced by hydrogels^62,70,79^ and (degradants from) PLGA scaffolds.^85^

*Dorsal Root Ganglia (DRGs)* have been studied for neuritogenesis^10,82^ and have been combined with spinal cord-derived motor neurons (MNs) in rat models of neuromuscular interfaces.^73^ However, this last study demonstrated that enhanced axonal outgrowth by DRGs is inferior to a mixture of sensory and motor cells, that can generate responding muscles with enhanced electrophysiological recovery and axon maturation.^73^

### External factors for innervation

**Figure 9.**
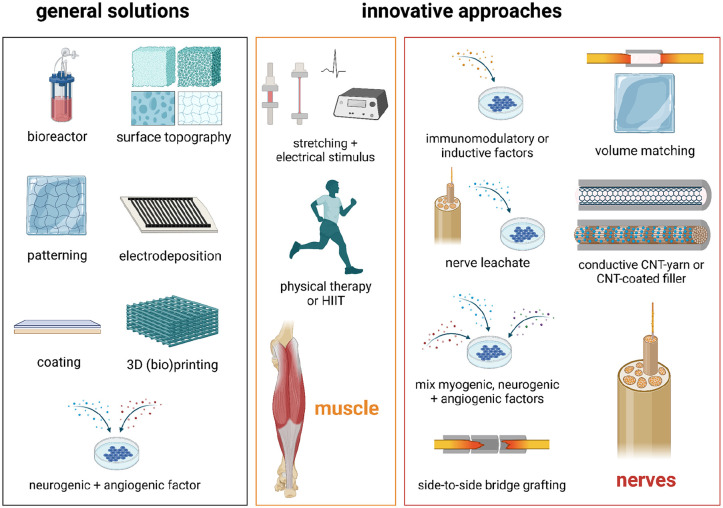
Schematic representation of general and innovative approaches with use of external factors for reconstruction of nerves (red) or muscle (orange).

The effectiveness of a tissue engineering approach relies on the applied biomaterial and cells, as well as on external stimuli of biological, chemical, or physical nature (Table 5).^17,28^ Biological stimuli include the addition of bioactive molecules to media,^53^ polymers,^15,20,69,73^ coatings,^18,22^ or via genetic modification,^37,89^ and are most commonly applied to mediate cell-cell and cell-material interactions for controlled cell behavior and tissue repair.^150^ Chemical stimuli often involve optimization of polymer composition, filling^77,119^ and coating.^16^ Physical stimuli typically include modification of biomaterial geometry,^78^ biomechanical properties^151^ and exposure to stress or compression,^23^ but for nerve signal transmission also include electrical conductivity,^152^ electrical stimulation^25^, and conductive coatings.^25,55,56^ A number of external factors have been studied in the included reports (Figure 9).

**Table 5.** Studied innervation inducing external stimuli with the (biological, chemical, physical) origin, disease model, application form, host, their effect on cell and tissue, and their combined application with biomaterials and cells. Their cellular inducing effect is indicated as high promotion (++) or moderate promotion (+).

External stimulus	Origin	Applied in	Form	Host	Neuro-genesis	Myo-genesis	Angio-genesis	Cell attachment	Cell alignment	Recover function	Biomaterial		Cells
BDNF^53,55,59,62,70,73,89,114^	Biological	Bone Cornea Sciatic nerve Skeletal muscle Skin	Culture medium Biomaterial incorporation Gene modification	Mouse Rat	++		++		++		ND	AGA CH COL DA FIB GEL HA	AD-MSC BM-MSC CSK FB HDMEC HUVEC iPSC / iPSC-NC MN PSM SC SN
CNTF^55,73^	Biological	Sciatic nerve Skeletal muscle	Biomaterial incorporation Culture medium	Rat	++		+				ND	AGA GEL	iPSC MN PSM
GDNF^53,55,73,89,104,114^	Biological	Cornea Sciatic nerve Skeletal muscle Skin	Culture medium Biomaterial incorporation Gene modification	Dog Rat	++		+ (skin)				ECM ND	ANG AGA COL DA GEL HA	AD-MSC CSK FB HDMEC HUVEC iPSC / iPSC-NC MN PSM SC SN
NGF^53,54,65,69,73,82,89,91^	Biological	Sciatic nerve Skin Subcutaneous	Culture medium Biomaterial incorporation Gene modification	Mouse Rat	++		++				ND SYN	AGA CH COL FB GEL hp-HA PCL	AD-MSC DRG FB HDMEC HUVEC KC MN SC SN UDSC
NT-3^53^	Biological	Skin	Culture medium		++		+				ND	COL	FB HDMEC HUVEC
CT-1^73^	Biological	Sciatic nerve	Biomaterial incorporation	Rat	++						ND	AGA GEL	MN
IGF^69^	Biological	Subcutaneous	Biomaterial incorporation	Mouse		++					ND	hp-HA	UDSC
LN^14,20,22,62,70,73,82^	Biological	Sciatic nerve	Biomaterial coating Biomaterial incorporation	Rat	++			++	++		ND SYN	AGA CH COL FIB GEL PCL	BM-MSC DRG MN SC
FN^20^	Biological	Sciatic nerve	Biomaterial incorporation	Rat				+	++		ND	CH COL	BM-MSC SC
PDL^22^	Biological	Sciatic nerve	Biomaterial coating	Rat				++			SYN	PCL	BM-MSC
CH^18^	Biological	Skin	Biomaterial coating		++			++			ND	ALG	BM-MSC
FKN^15^	Biological	Sciatic nerve	Biomaterial incorporation	Rat	++						SYN	AGA PSF	
VEGF^65,69,74^	Biological	Sciatic nerve Subcutaneous	Culture medium Biomaterial incorporation Gene modification	Mouse Rat			++				ND	COL FB GEL HA	DRG UDSC
NL^63^	Biochemical	Sciatic nerve	Culture medium	Rat	++					++	ECM	ANG	AD-MSC
ACC^64^	Biochemical	Larynx	Culture medium	Rat						++	ND	COL	AD-MSC MPC
AGR^64,71^	Biochemical	Larynx Pelvic floor muscle	Culture medium	Rat		++					ND	COL FB GEL HA	AD-MSC MPC
Carbon nanotubes^16^	Chemical	Sciatic nerve	Covalent linking	Rat	+						SYN	PLDLA	DRG
Plastic compression^92^	Physical	Bladder	Hydrogel compression	Rat							ND	COL	AD-MSC SMC
Mechanical stretching^26^	Physical	TA muscle	Cyclic construct stretching	Rat	+						ECM	BAM	MPC
Electrical Pulse^25^	Physical	Peripheral nerve	Metal biomaterial coating		++			+ (LN)	++		ND	SF	
Micropattern^25,55,56^	Physical	Peripheral nerve Skeletal muscle	Biomaterial modification		++	++			++		ND SYN	GEL PA SF SIL	AD-MSC CMC iPSC PSM
Electrodeposition^87^	Physical	Peroneal nerve	Metal biomaterial coating	Rat	++					++	SYN	AGA PEDOT	
Electrospinning^21,22,70,79,86,90,96^	Physical	Muscle Pudendal nerve Sciatic nerve	Biomaterial alignment	Rat	++				++		ND SYN	CH dECM FIB PCL cYarn	AD-MSC BM-MSC DRG MD-MSC MPC SC
3D printing^10,24,66,71,94,114^	Physical	Bone Cornea Cranial bone Pelvic floor muscle Sciatic nerve TA muscle	Biomaterial alignment	Rat	++				++		ND SYN	DA FB GEL HA LMS-GelMA PCL ß-TCP	AD-MSC CSK DRG HUVEC iPSC-NC MPC RUVEC SC
HIIT^61^	Physical	TA muscle	Physical exercise	Rat	++		++			++	ECM	dAM	AD-MSC
Physical therapy^60^	Physical	Skeletal muscle	Physical exercise	Human			++			++	ECM	BAM	
Side-to-side bridge^72^	Physical	Tibial nerve	Surgical procedure	Rat	++						ECM	ANG	
Perfusion based bioreactor^95^	Physical	Subcutaneous	Culturing method	Rat							ND	COL	SVF SkMb

#### Biological stimuli

Within the group of biological stimuli, neurotrophic factors that have often been applied for innervation include nerve growth factor (NGF), glial cell line-derived neurotrophic factor (GDNF), brain-derived neurotrophic factor (BDNF), neurotrophin-3/4/5 (NT-3/4/5), ciliary neurotrophic factor (CNTF) and neuropoietic cytokines.^37^ These factors have been tested in rodent animal models, and GDNF had additionally been examined in dogs.

*NGF, GDNF, BDNF, CNTF* and *NT-3* are widely studied *neurotrophic factors*. NGF promotes neurogenesis and can be added to biomaterials,^54^ or culture medium of neuronal cells.^65,82^ NGF can also be cross-linked with genepin to conduits, which has demonstrated to improve histology and function of rat peripheral nerves.^91^ NGF and other neurotrophic factors are often combined. As such, a mix of insulin-like growth factor (for myogenesis), NGF and FGF (for neurogenesis), and VEGF (for angiogenesis), has demonstrated to enhance cell viability, blood vessel formation, and nerve growth into transplants.^69^ Furthermore, gene modification by CRISPRa can be used to boost expression of NGF, BDNF and GDNF to enhance myelination, nerve regeneration and recovery in rats.^89^ These factors and NT-3 have been shown to also possess angiogenic potential.^53^ Furthermore, together with CNTF, they have been tested on aggregates of spinal motor neurons.^73^ BDNF has demonstrated effective for muscle and bone,^55,59^ and for in vitro innervation of cornea when combined with GDNF.^114^ Innervation can also be achieved by BDNF-mimicking peptide with laminin-derived motifs that enhance BDNF, CNTF and NGF-secretion,^62,70^ or by injection of GDNF into ANG to further improve functional recovery of rat sciatic nerves.^104^

*Laminin (LN)* and *fibronectin (FN)* are *ECM inductive molecules* that influence nerve growth.^153^ LN is found in the basal lamina (even after decellularization),^14,154^ and can be used as a coating to promote cell adhesion.^22,73^ In vitro, mouse neurites show a preference for mouse-derived LN^14^ and polymerized LN can enhance neurite growth,^82^ but monomeric LN is beneficial for functional nerve regeneration under in vivo conditions.^82^ Although a FN and LN coating can respectively improve BM-MSC and SC survival, SCs on FN coating showed a superior functionality in vivo.^20^

*Fractalkine (FKN)* and *Chitosan (CH)* are *immunomodulatory molecules*.^15,18^ FKN aids in nerve repair,^15^ whereas modification by concentration-based CH coating can control cell behavior in innervation.^18^

*VEGF* is an *angiogenic factor* that indirectly influences nerve growth in rats by targeting non-neuronal cells.^74^

#### Chemical stimuli

(Bio)chemical agents have extensively been tested for innervation in rats and are typically added to culture media or used for modification of a biomaterials.

*Nerve leachate (NL)* is a natural mix of *neurotrophic factors* that has been reported to improve histology and tissue function when added to culture medium.^63^

*Acetylcholine (ACC), agrin (AGR)* and *neuregulin (NRG)* are *inductive factors*.^64^ AGR enhances functional muscle regeneration and formation of ACC-receptor clusters, and accelerates innervation of pelvic floor muscle in rats.^71^ A mix of ACC, AGR, and NRG in media can enhance innervation and regeneration of rat larynx by inducing expression of motor endplates.^64^

*Carbon nanotubes (CNTs)* are *conductive factors* that have shown successful as conduit fillers for repair of rat sciatic nerves.^16^

#### Physical stimuli

Physical stimuli often indirectly modify properties of cells, biomaterials, and constructs through force application, biomaterial manufacturing, or physical exercise.^155,156^

##### Biomaterial modification

Cell behavior is influenced by the surface topography of a biomaterial (e.g. grooves, fiber alignment, pores, and roughness), which can be modified to direct and align nerve cells.^25^ Common approaches include micropatterning and electrodeposition.^29,87,96,108,157^ For example, myofibrillar or ECM proteins can be mimicked with patterns of around 10 μm in size to help adherence and alignment of myotubes.^55^ Likewise, patterned hydrogels can steer cell migration and differentiation, by using cell preferences to a specific material stiffness.^56^ Furthermore, electrodeposition allows integration of conductive patterns (with electrical stimulation),^25^ that can boost mechanical strength without physical hindrance of axonal elongation.^87^

With advanced techniques (e.g. electrospinning and 3D (bio)printing) also the completely redesign of biomaterials is possible.^29,108,125,135,158^ Electrospinning of a 3D structure can be used to mimic the hierarchical organization and biological function of ECM,^79^ and aligned nanofibers can promote nerve regeneration in vivo, with faster axon regrowth and motor function recovery.^70,79^ Furthermore, electrospun PCL has demonstrated innervation^22,86,90,96^ of muscle flaps,^96^ and of nerves.^90^ At the same time, electrospinning of CNTs allows control over electrical conductivity, biocompatibility and flexibility, to aid in nerve regeneration.^21^ 3D bioprinting allows creation of bioengineered structures with precise placement of various cell types and bioactive factors.^66^ This has been applied for 3D-printed skeletal muscle for rapid integration of rat neural networks after implantation, with fast restoration of function and improved regeneration.^66,71^ Bioprinted cranial bone can support long-term cell survival, exhibits angiogenic capability and encourages neurogenetic differentiation of neural cells and osteogenic differentiation of MSCs.^94^ 3D printing further allows fabrication of customized nerve conduits^10^ or in vitro cornea.^114^ It has further been shown that 3D-printed and randomly porous β-TCP both support expression of neural and angiogenic growth factors in SCs, but only 3D-printed scaffolds support growth of SCs with a normal morphology.^24^

##### Electro- and biomechanical forces

Perfusion-based bioreactors to control flow rates,^95^ and electrical impulses to control axonal alignment and outgrowth,^37^ are other well-known examples of physical stimuli. For muscle cells, electrical stimulation has been combined with cyclic stretching prior to implantation, and showed crucial for muscle contractions and recovery of volumetric muscle loss (VML) in rats.^26^ Moreover, geometrical matching of implants to injury sites can increase functional recovery,^26,159^ and volume matching of compressed collagen to the rat bladder wall can enhance regeneration and reconstruction.^92^

##### Physical exercise

Recent innovative treatments have incorporated physical therapy and exercise. High-intensity interval training has been shown to enhance vascular ingrowth and healing after transplantation of human decellularized amniotic membrane for treatment of VML injury.^61^ In a clinical study, physical therapy improved the muscle function in three-fifths VML patients.^60^

##### Surgical techniques

Modified surgical techniques can be used to enhance innervation. In side-to-side bridging, an acellular nerve graft is sutured end-to-side to an epineurial window of a parallel host nerve, to enhance ingrowth of host neurons into the graft.^72^

### Risk of bias and quality assessment of included studies

Assessment of quality and risk of bias were separately conducted for in vitro (Supplemental Figure S2) and in vivo studies (Supplemental Figure S3). In vitro studies showed a high to moderate risk of bias.^52^ Studies rated well on methodology, outcome measures, and presentation of results, with clear objectives, control groups, and statistical analysis. However, they lacked operator details, sample size justification, sampling technique, randomization processes, and blinding of assessors. The NIH checklist indicated a clear research question, study population, pre-experimental measurements, experiment duration, and consistent exposure for in vitro studies. However, some concerns were raised, as most studies lacked sample size justification, blinding of assessors, measurement of confounders, and repeated exposures. The JBI-Ana checklist indicated a clear study population, exposure and outcome measurements. However, studies lacked identification of and strategies for confounders, sample inclusion, and an appropriate statistical analysis. Despite these concerns about the risk of bias, the overall quality of in vitro studies was moderate to good.

In vivo studies showed a moderate risk of bias. They rated well on control, follow-up and representativeness, but they were self-reported and lacked initial measurements, blinding of assessors, and statements on loss during follow-up. Some quality concerns were raised by the QUADAS-2 and JBI-Exp tools. The QUADAS-2 tool indicated clear inclusion criteria, measurements, and reference representativeness, but research questions were unclear and groups were inconsistent (analysis on sub-group of animals and use of different references). According to the JBI-Exp tool, studies rated well on cause-effect description, treatment consistency, controls, outcome measures, and statistical analysis. However, they lacked consistent group sizes, follow-up, and initial measurements. Despite some risk of bias, the overall quality of in vivo studies was moderate to good.

## Discussion

Various treatment options are available for injury of organs and peripheral nerves, but an ideal solution is still absent in terms of recovery and regeneration. Autografts and allografts are the first choice of treatment, but tissue survival and functionality are endangered by their limited vascular and neural network. In the last decade, tissue engineering has received great attention and evolved rapidly to address these problems.^29,45,160^ Unfortunately, despite the new hope they offer, clinical translation of these tissue-engineered solutions is not always achieved. Previous reviews have extensively studied specific aspects of tissue engineering for innervation (e.g. specific organs, state-of-the-art approaches, or parts of the nervous system).^45,108,161,162^ However, to our knowledge, the interplay between biomaterials, cells and external stimuli,^17,163^ or solutions for multiple organs have not been studied. Considering the unknown nature of these areas, we performed a systematic review on all elements of the tissue engineering triad for innervation of all organs and peripheral nerves. The aim of this review was to identify high-potential solutions and unexplored areas, that might help bridge the gap toward clinical translation.

Of the 3626 identified articles, 68 studies were included. Quality assessment with multiple tools resulted in exclusion of 291 low-quality reports with a suitable topic, thereby potentially removing relevant and recent publications from this review. For example, recent articles by Kim and Kim^164^ and Rousseau et al.^165^ on muscle innervation, were not included due to the absence of ethical approval and incomplete description of test subject conditions, respectively. Many studies reported poorly on methodology, including the sample size, technique, randomization, and blinding. Included studies rated moderate to good on risk of bias. Remarkably, only one clinical study was retrieved from the systematic search. This emphasizes the troublesome and challenging road between in vivo proof-of-principle and actual clinical translation.^166^

### Biomaterial

An ideal biomaterial for tissue regeneration and innervation is considered to closely resemble native tissue in their properties.^37,43,167,168^ For instance, its structure should mimic the local host architecture,^169–171^ and incorporation of bioactive molecules can provide inductive signals for cells.^150,172,173^ Natural polymers resemble some aspects of native tissue (e.g. biomechanical properties and some signaling cues) but require modifications to form structurally stable scaffolds.^66,74,75^ To this end, recent methods have applied polymer mixing, electrospinning, plastic compression, 3D (bio)printing, electrical stimulation, and incorporation of inductive signals.^29,43,174^ Of the studied natural-derived biomaterials, chitosan, collagen, and silk fibroin demonstrated encouraging outcomes in nerve regeneration^20,25,62,70,76,78,80,83,91,98^ and innervation of various organs (e.g. bladder,^92,101,102^ bone,^59^ larynx,^64^ esophagus,^75^ and urethra^99^). However, direct comparison was impossible, as the same organ (with the exception of the sciatic nerve) is at best studied thrice. Natural-derived biomaterials have been studied in small animals and pigs.

Synthetic materials are easily customized but their biodegradability is challenging for regeneration of soft tissue,^21,93,136^ and for some hard tissues like bone.^56^ Furthermore, their breakdown products can cause considerable adverse effect for cells and the host.^17,108,163^ Of the synthetic materials, PCL was often studied and demonstrated encouraging results for regeneration of pudendal and sciatic nerves.^10,22,82,84,86,90^ Synthetic materials have been tested in small animals and macaques for innervation.

ECM-derived biomaterials perfectly resemble native tissue in hierarchical structure,^157,170,175^ and exhibit outstanding properties for cell induction.^29,176,177^ Despite these promising properties, they are seldomly applied for tissue innervation. This is likely related to their restricted retrieval and the adverse effects seen when used across species (xenogeneic).^13,14^ Of the ECM-derived biomaterials, acellular nerve grafts (ANGs) from xenogeneic or allogenic sources were widely studied and showed promising outcomes for regeneration of sciatic and tibial nerves in rats.^13,14,63,68,97^

Selection of a biomaterial involves weighing of their unique advantages and limitations for effective application and clinical use. However, as seen in this review, most innovative biomaterials do not reach clinical trials. Why does this happen? Most research relies on funding and grants, that permit short-term studies. Even with successful outcomes funding for followup research may proof difficult to obtain, due to extreme competition for the same grants, fast development of exciting new research fields or even simply through bad luck, for instance in case of new national or international cash flow restrictions toward research due to economical changes. Furthermore, in vitro studies are typically followed by in vivo studies in rodents. This is where most research ends due to vascularization issues during upscaling, bad representation of the human body by rodent models or difficulties to acquire ethical approval for clinical studies or large animal models. For example, current clinical treatment of nerve lesions and tissue innervation are limited to small constructs (<3 cm),^41^ and tissue volumes.^42^ Moreover, our review findings supportively demonstrate the inability to access biological material from humans or even large animals. Although many promising applications were presented, most studies did not discuss or consider how these future roadblocks could be overcome to bridge the current gap toward clinical application.

In clinical setting several FDA-approved, artificial nerve conduits have demonstrated satisfying recovery but with significant side effects or regeneration incapability.^41^ Much, if not most, of these issues originated from failure to mimick native tissue composition and structure, and incompatibility due to cross-species use of biomaterials (xenografts). These problems could be resolved with constructs that closely mimic native tissue, that are capable of innervation over large volumes, and do not pose risk of immunorejection. For all these criteria, human-derived acellular matrices and their bio-inks form a promising biomaterial of choice. Acellular nerve grafts (ANGs) carry great potential as they have demonstrated efictive repair,^13,14,63,68,72,97,104^ with autograft-like results.^63,68^ In addition, denervated tissues are promising for future clinical applications, as they can outperform synthetic materials.^67^

### Cells

For innervation, a large variety of cell types are available, of which mainly stem-, progenitor- and differentiated cells have been examined.

Induced pluripotent stem cells (iPSCs) can differentiate into various cell lineages, but issues with availability and ethics limit their use.^11,178,179^ Nonetheless, innervation was reported to be more efficient for iPSCs-derived neural crest cells than for further differentiated Schwann cells.^11^

Mature stem cells are able to differentiate into multiple cell types, but their use is challenged by their specificity and limited availability.^140,180,181^ Although adipose- or urine-derived stem cells are easily accessible types,^9,61,63,69,89^ they rely on other cells for sufficient efficacy.^92^ Muscle progenitor cells share this limitations.^26,71,86^ Furthermore, stem cells can be derived from tissues like muscle^57,96^ or bone marrow,^18,20,22,57–59,97,100^ but this requires an invasive retrieval procedure.^182,183^ Bone marrow-derived stem cells excel in promoting innervation,^59,97,100^ but are under certain conditions outperformed by tissue-specific cell types.^20,57,58^

Bone marrow mononuclear cells (BM-MNCs) are easily retrieved from blood, but pathogen removal from their cell cultures and their standardization are difficult.^98^

Alternatively, mature neural cells (e.g. neurons, glial- and Schwann cells) can be used for innervation. Neurons accelerate functional recovery as they prevent the need for extensive host neuron growth within a biomaterial.^37,73^ Glial cells guide and attract neurons by secretion of neurotrophic factors,^24^ and they boost neural regeneration by immunomodulation and removal of debris.^137,139^ Although the clinical applicability of neural cells is challenged by their limited availability,^42,93^ they can also be derived from stem/progenitor cells.^11,137^

Selection of one or more cell types involves weighing of preferences and drawbacks. Our review demonstrates that autologous cells are not within the scope of current research. However, in terms of current achievements and clinical potential, cell-seeding of patient-retrieved, autologous cells before implantation holds undeniably great promise. This has extensively been reported to be successful for scaffold functionalization (i.e. to promote innervation).^9,184,185^ The need for more innovative approaches that are based on autologous cell-seeding, is indicated by current failure of biomaterials to mimick tissue function in absence of cells and by their immunocompatibility in presence of allogenic or xenogeneic cells. As these issues, amongst others, hold back clinical translation, research could greatly benefit from development in this area. Especially innovative research with autologous mature neural cells are promising for future clinical applications, as they can be directly extracted or derived from stem/progenitor cells and both accelerate recovery and enhance regeneration.^24,37,73,137,139^

### External stimuli

Many constructs rely on external stimuli for their performance.^155,171,186^ Biological and chemical stimuli are vital for cell-cell and cell-material interactions.^155,187^ For innervation, these have been added to culture media, embedded in scaffolds, or applied as coatings or for alteration of gene expression, either as neurotrophic factors, ECM-derived proteins, or other bioactive/chemical compounds.

Remarkably, nerve growth factor (NGF), brain-derived neurotrophic factor (BDNF), neurotrophin-3 (NT-3), and glial cell line-derived neurotrophic factor (GDNF), are neurotrophic agents that have been found to aid neurogenesis as well as angiogenesis,^53,162^ while the vascular endothelial growth factor (VEGF) is an angiogenic factor that also encourages neurogenesis.^74^ Mixtures of bioactive molecules have been found to outperform innervation of single neurotrophic factors, by promoting neurogenesis, myogenesis, and angiogenesis.^69^

In terms of physical stimuli, innervation has been enhanced by conductive materials combined with electrical stimulation,^25,87,108^ and by physical therapy to promote muscle regeneration.^60,61^

The included reports on external stimuli emphasize the importance of tissue functionality and body dynamics for successful innervation. Although a range of external stimuli have been examined, many promising solutions remain relatively unexplored. In that regard, future research could benefit from advancements like exertion of biomechanical forces, polysurgery technique, and new conductive coatings.

### Solutions to current limitations

Nowadays, a wealth of tissue engineering solutions has been reported for innervation of peripheral nerves and tissues.^29,38,43^ Herein cell-seeding with autologous cells is desired for biomaterial functionalization, as no gradual cell replacement by host-derived cells is required during regeneration,^103^ while preventing immunorejection associated with the use of allogenic cells.^163,188–191^ Furthermore, extracellular matrix (ECM)- and natural-derived (ND) biomaterials contain valuable (bio)physical, chemical and geometrical cues for interaction with cells.^29,42,43,168,176^ This naturally offers cell-cell interactions even without a need for cell-seeding, that is expensive in terms of finances, time and resources, and thus clinically less appealing.^173,192,193^ Ideally, their manufacturing is time-managed to align with surgery,^194,195^ but safe storage of solutions like decellularized matrices and bio-inks for 3D-printed scaffolds is possible.

Furthermore, due to the complex interplay between aspects of the tissue-engineering triad, success of innervation relies on extensive collaboration of experts in the field of health care, stem cell research, biomaterial production, regenerative medicine, biomedical engineering, 3D bioprinting, and physics (amongst many more). Current hurdles encountered toward clinical application include cytotoxicity, poor mechanical properties, manufacturing difficulties, sterility concerns, costs, uncontrolled degradation rates, insufficient neuro-conductivity, or financial funding for research. To solve these issues, this field could benefit from inter-study comparison, allowing evolutionary advancement toward preferred solutions. Although the ideal tissue-engineered construct with vital properties for efficient innervation still needs to be designed, the rapid advancement of tissue-engineered solutions make for a bright future.

## Future perspective

Much research has been performed on tissue engineering for innervation of direct (sciatic) nerve repair. Innervation of organs and tissues (e.g. skin, bladder, larynx, esophagus, and urethra) is rather unexplored and thereby forms great opportunity for future research. For instance, considering the importance of sensation for reproductive organs such as the vagina, innervation-based solutions would especially be valuable in this area. We covered in our previous publications how current clinical issues in vaginoplasty could be resolved by tissue-engineered neovaginas,^28,154^ for which innervation is crucial to achieve full or even satisfactional functionality.

Today, most research involves cross-species application of cells (or biomaterial), thereby complicating the step toward clinical translation. Future developments could greatly benefit from use of autologous cells, as they induced biomaterial functionalization without risk of immunorejection.

More exploration of ECM-derived and ND-biomaterials could offer innovative solutions in the future, as (bio)physical, chemical and geometrical cues are naturally present for required cell-material interaction. These biomaterials also closely resemble native tissue in terms of biomechanical, geometrical and chemical composition, and pose no risk of immunorejection. Thereby forming the biomaterials most likely to fulfill the clinical demands.

Lastly, with a future focus on upscaling to human-relevant sizes, it would become possible to offer solutions beyond the current limit of 3 cm to treat large nerve injuries.

## Conclusion

The human body is a complex system, requiring innovative solutions for tissue-engineered regeneration and innervation. Although many methods are available, a distinctive gap complicates research from finding translation to clinical settings. To bridge this gap, future research should focus on ECM-derived and ND-biomaterials, and autologous cell seeding for scaffold functionalization. Moreover, to overcome current limitations and fulfill clinical demands, innervation of larger biocompatible constructs is needed that are capable of satisfactional recovery without side effects or risk of regeneration failure.

## Supplemental Material

sj-docx-1-tej-10.1177_20417314251316918 – Supplemental material for Advances in tissue engineering of peripheral nerve and tissue innervation – a systematic review Supplemental material, sj-docx-1-tej-10.1177_20417314251316918 for Advances in tissue engineering of peripheral nerve and tissue innervation – a systematic review by Jayson Sueters, Rowan van Heiningen, Ralph de Vries, Zeliha Guler, Judith Huirne and Theo Smit in Journal of Tissue Engineering

sj-docx-2-tej-10.1177_20417314251316918 – Supplemental material for Advances in tissue engineering of peripheral nerve and tissue innervation – a systematic review Supplemental material, sj-docx-2-tej-10.1177_20417314251316918 for Advances in tissue engineering of peripheral nerve and tissue innervation – a systematic review by Jayson Sueters, Rowan van Heiningen, Ralph de Vries, Zeliha Guler, Judith Huirne and Theo Smit in Journal of Tissue Engineering

sj-docx-3-tej-10.1177_20417314251316918 – Supplemental material for Advances in tissue engineering of peripheral nerve and tissue innervation – a systematic review Supplemental material, sj-docx-3-tej-10.1177_20417314251316918 for Advances in tissue engineering of peripheral nerve and tissue innervation – a systematic review by Jayson Sueters, Rowan van Heiningen, Ralph de Vries, Zeliha Guler, Judith Huirne and Theo Smit in Journal of Tissue Engineering
