# Supplementary material for: Advances in tissue engineering of peripheral nerve and tissue innervation – a systematic review
Source: J Tissue Eng. 2025 Feb 5;16:20417314251316918. doi: 10.1177/20417314251316918 (PMC11795627; doi:10.1177/20417314251316918)
Supplement: sj-docx-1-tej-10.1177_20417314251316918 – Supplemental material for Advances in tissue engineering of peripheral nerve and tissue innervation – a systematic review [file sj-docx-1-tej-10.1177_20417314251316918.docx]

Supplementary material S1

PubMed Session Results (02 Nov 2023)

| **Search** | **Query** | **Results** |
| --- | --- | --- |
| #3 | **#1 AND #2** | 520 |
| #2 | **innervat*[tiab] OR reinnervat*[tiab]** | 70,113 |
| #1 | **"Tissue Engineering"[Mesh] OR "Tissue Scaffolds"[Mesh] OR "Guided Tissue Regeneration"[Mesh:NoExp] OR "tissue engineer*"[tiab] OR "tissue regenerat*"[tiab] OR "tissue scaffold*"[tiab] OR "tissue graft*"[tiab]** | 105,909 |

Embase.com Session Results (02 Nov 2023)

| **Search** | **Query** | **Results** |
| --- | --- | --- |
| #4 | **#3 NOT ('conference abstract'/it OR 'conference review'/it)** | 2,154 |
| #3 | **#1 AND #2** | 2,393 |
| #2 | **'innervation'/exp OR 'reinnervation'/exp OR innervat*:ab,ti,kw OR reinnervat*:ab,ti,kw** | 135,576 |
| #1 | **'tissue engineering'/exp OR 'tissue scaffold'/exp OR 'tissue graft'/exp OR 'tissue engineer*':ab,ti,kw OR 'tissue regenerat*':ab,ti,kw OR 'tissue scaffold*':ab,ti,kw OR 'tissue graft*':ab,ti,kw** | 326,962 |

Web of Science (Core Collection) Session Results (02 Nov 2023)

| **Search** | **Query** | **Results** |
| --- | --- | --- |
| #3 | **#1 AND #2** | 468 |
| #2 | **TS=("innervat*" OR "reinnervat*")** | [71,](https://pubmed.ncbi.nlm.nih.gov/?term=%22Duodenal+Obstruction%22%5BMesh%3ANoExp%5D+OR+%22Annular+pancreas%22+%5BSupplementary+Concept%5D+OR+%22Familial+duodenal+atresia%22+%5BSupplementary+Concept%5D+OR+%22duodenal+obstruction%2A%22%5Btiab%5D+OR+%22duodenum+obstruction%2A%22%5Btiab%5D+OR+%22duodenal+stenos%2A%22%5Btiab%5D+OR+%22duodenum+stenos%2A%22%5Btiab%5D+OR+%22duodenal+web%2A%22%5Btiab%5D+OR+%22duodenal+atresia%2A%22%5Btiab%5D+OR+%22annular+pancrea%2A%22%5Btiab%5D+OR+%22pancreas+annular%2A%22%5Btiab%5D&sort=date&ac=no)684 |
| #1 | **TS=("tissue engineer*" OR "tissue regenerat*" OR "tissue scaffold*" OR "tissue graft*"**) | 108,331 |

Scopus Session Results (02 Nov 2023)

| **Search** | **Query** | **Results** |
| --- | --- | --- |
| #3 | **#1 AND #2** | 484 |
| #2 | **TITLE-ABS("innervat*" OR "reinnervat*") OR AUTHKEY("innervat*" OR "reinnervat*")** | 81,618 |
| #1 | **TITLE-ABS("tissue engineer*" OR "tissue regenerat*" OR "tissue scaffold*" OR "tissue graft*") OR AUTHKEY("tissue engineer*" OR "tissue regenerat*" OR "tissue scaffold*" OR "tissue graft*")** | [132,](https://pubmed.ncbi.nlm.nih.gov/?term=%22Infant%22%5BMesh%5D+OR+%22Intensive+Care%2C+Neonatal%22%5BMesh%5D+OR+%22Intensive+Care+Units%2C+Neonatal%22%5BMesh%5D+OR+%22Neonatal+Nursing%22%5BMesh%5D+OR+infant%2A%5Btiab%5D+OR+newborn%2A%5Btiab%5D+OR+neonat%2A%5Btiab%5D+OR+prematur%2A%5Btiab%5D+OR+preterm%2A%5Btiab%5D&sort=date&ac=no)203 |
