# Supplementary figures and images for: Advances in tissue engineering of peripheral nerve and tissue innervation – a systematic review
Source: J Tissue Eng. 2025 Feb 5;16:20417314251316918. doi: 10.1177/20417314251316918 (PMC11795627; doi:10.1177/20417314251316918)

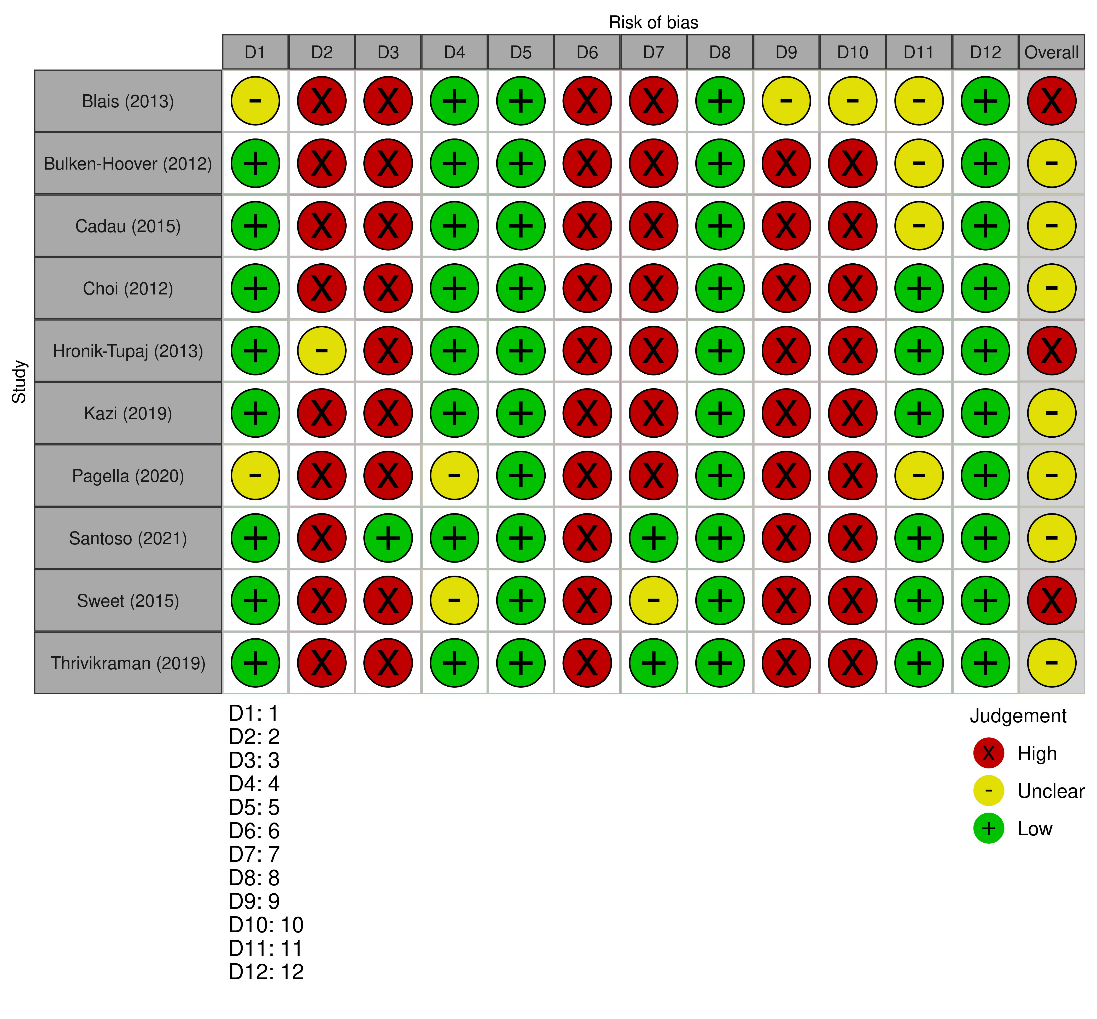

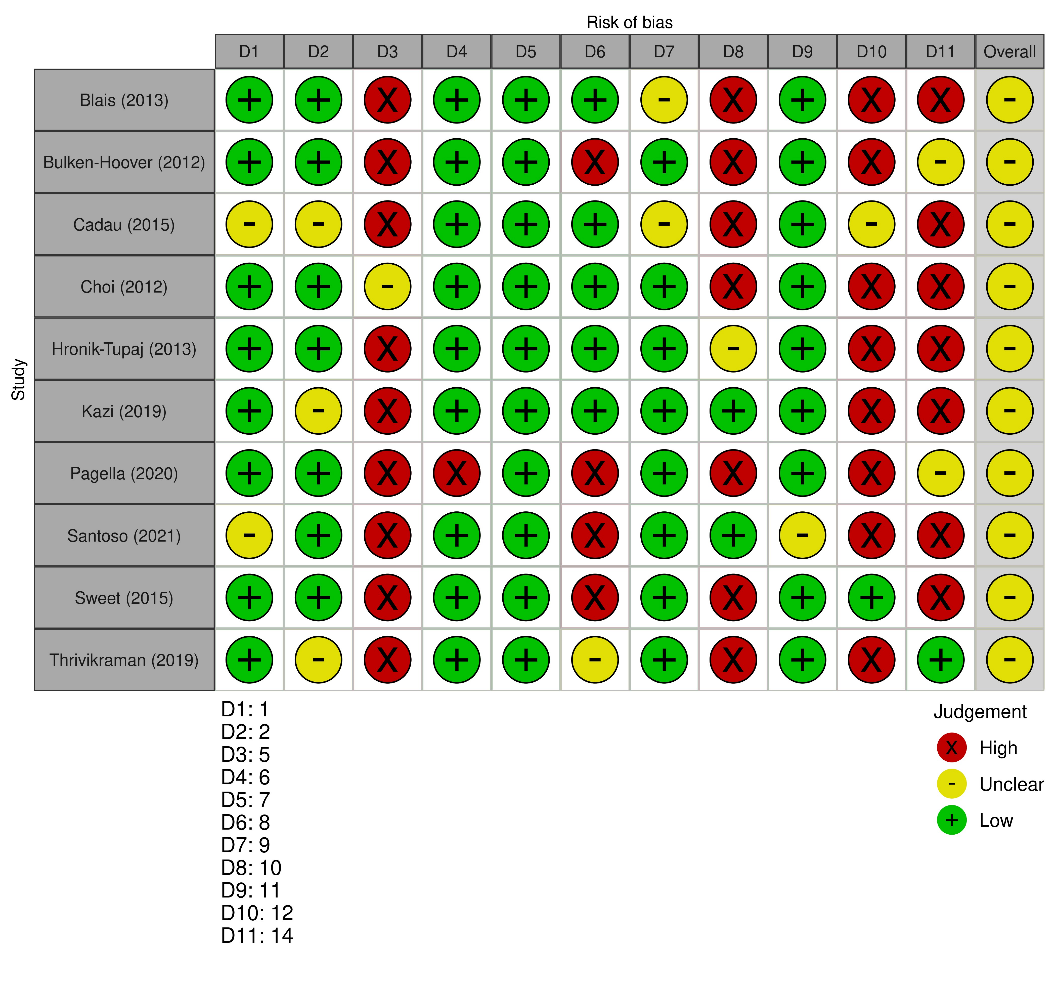


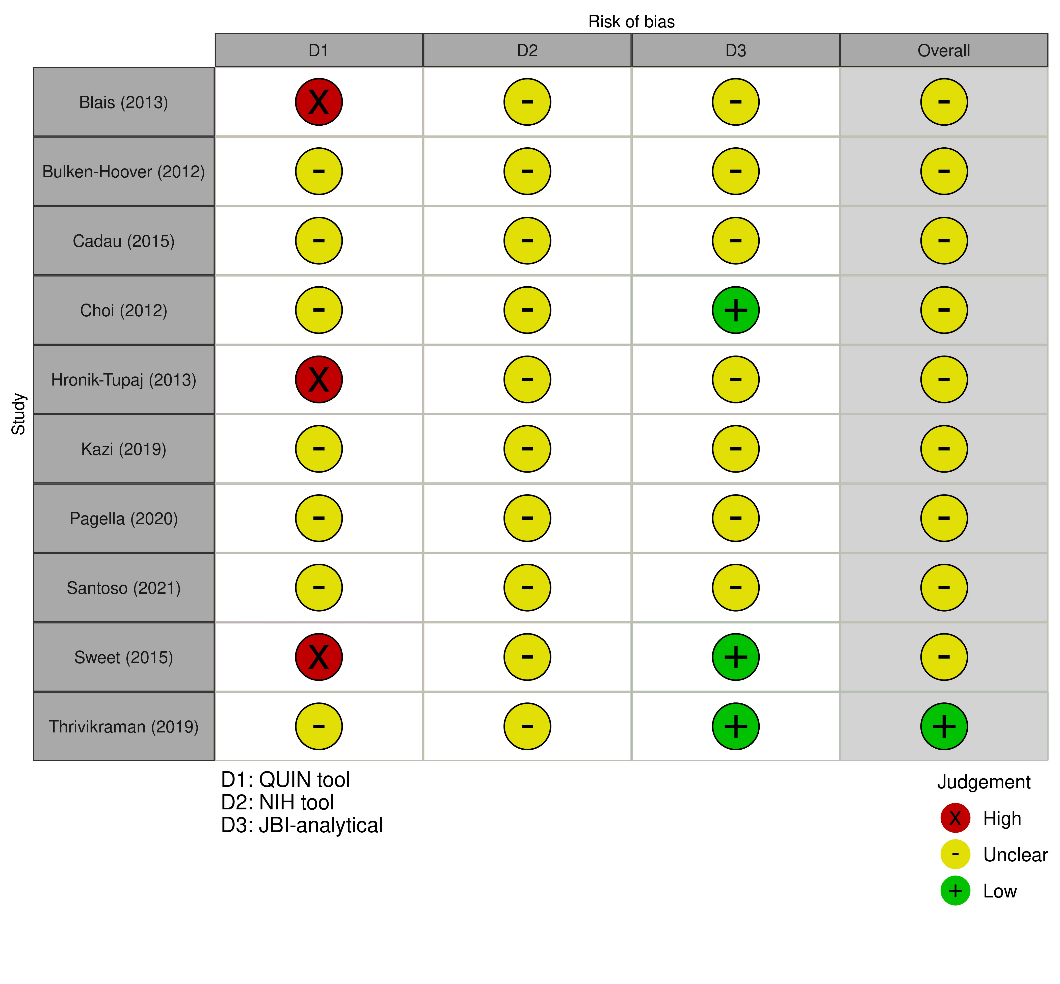

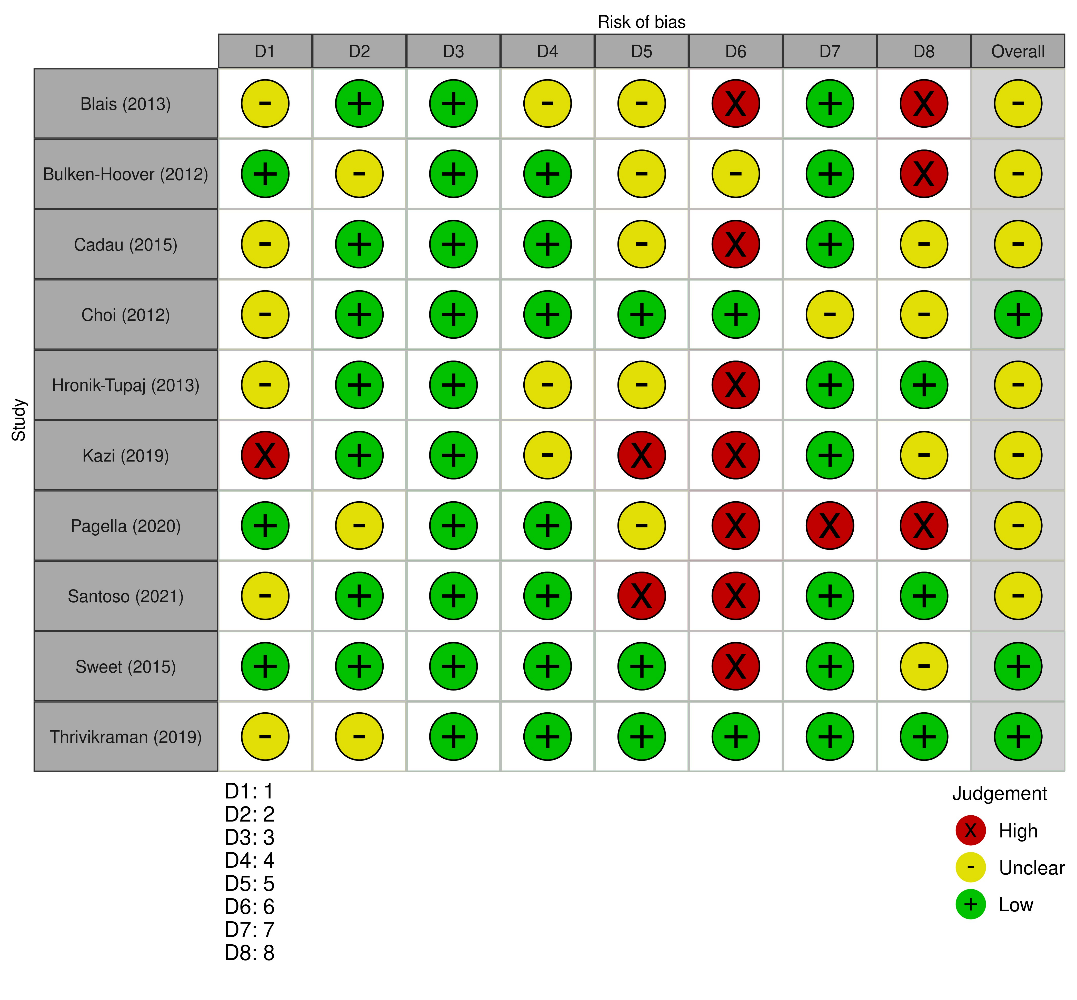


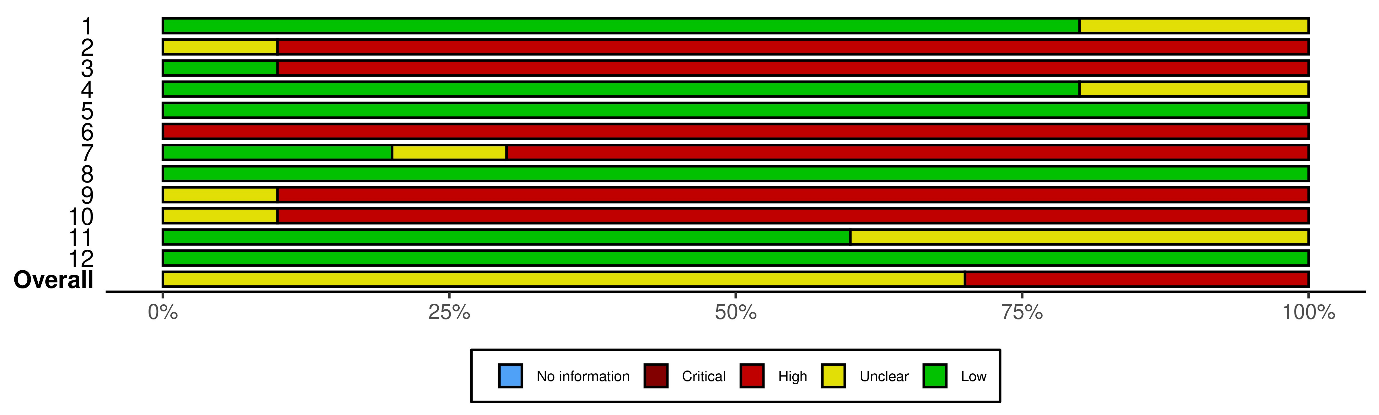


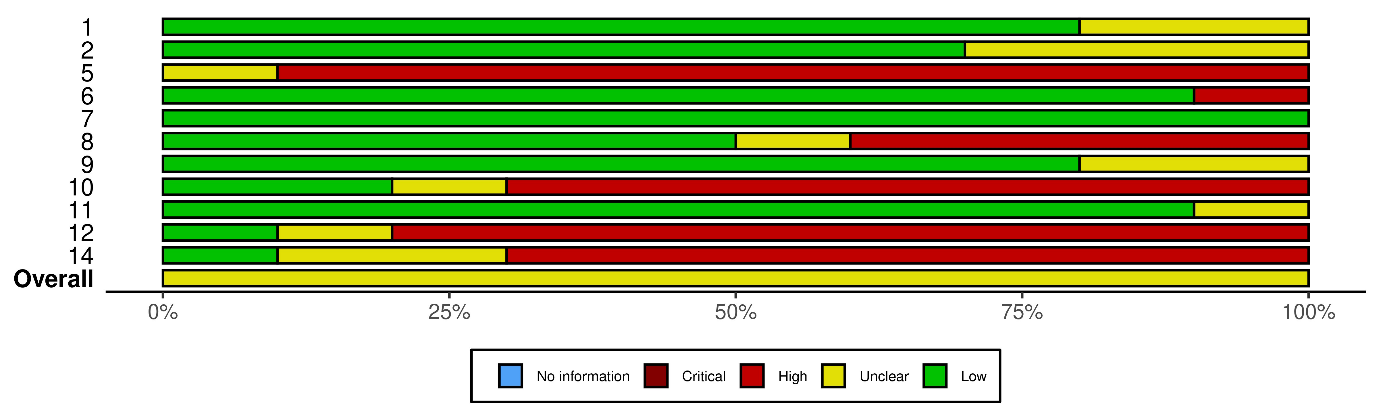


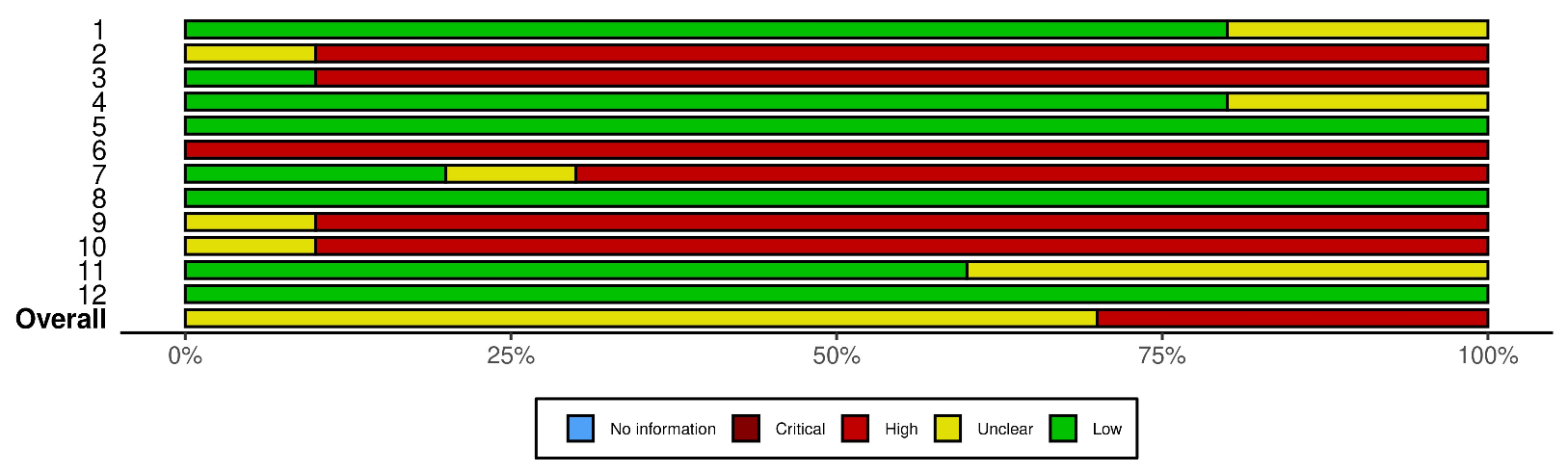

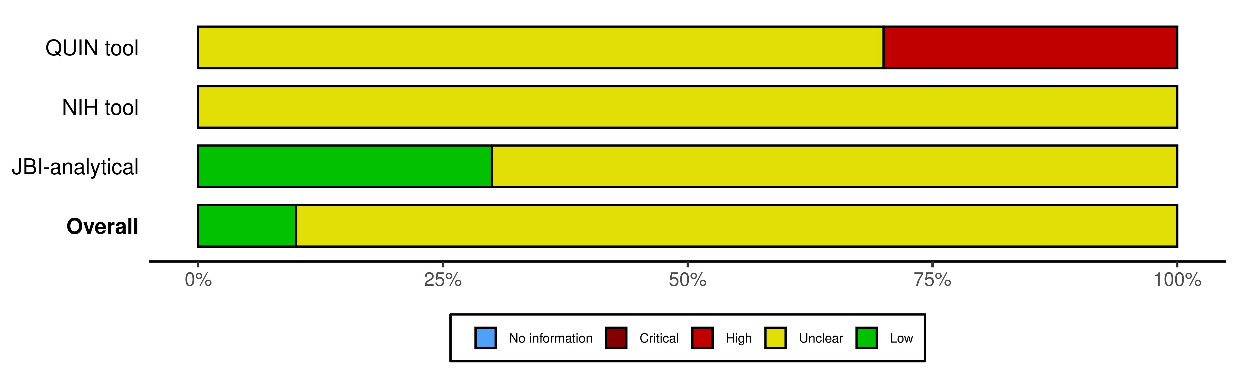

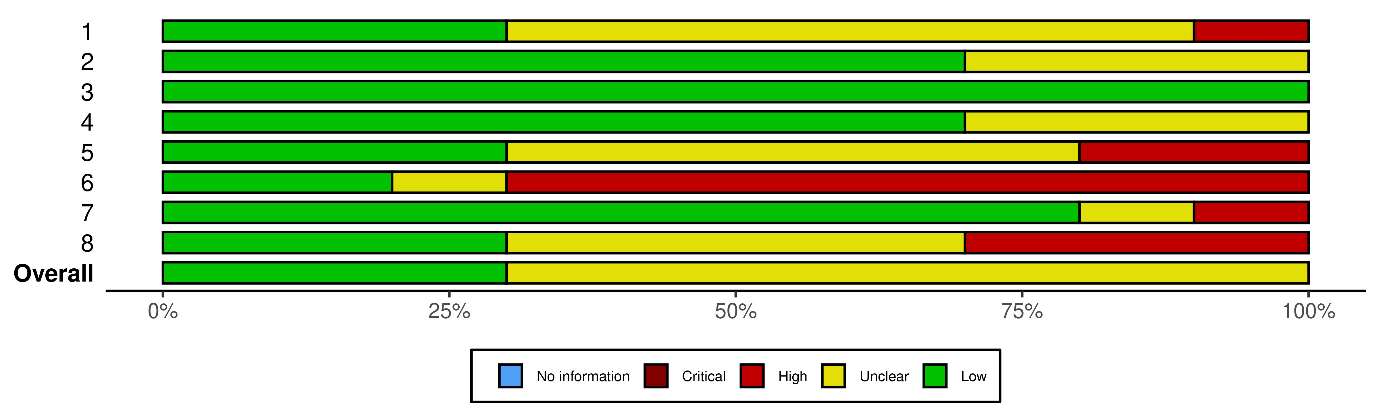

Supplement: sj-docx-2-tej-10.1177_20417314251316918 – Supplemental material for Advances in tissue engineering of peripheral nerve and tissue innervation – a systematic review [file sj-docx-2-tej-10.1177_20417314251316918.docx]

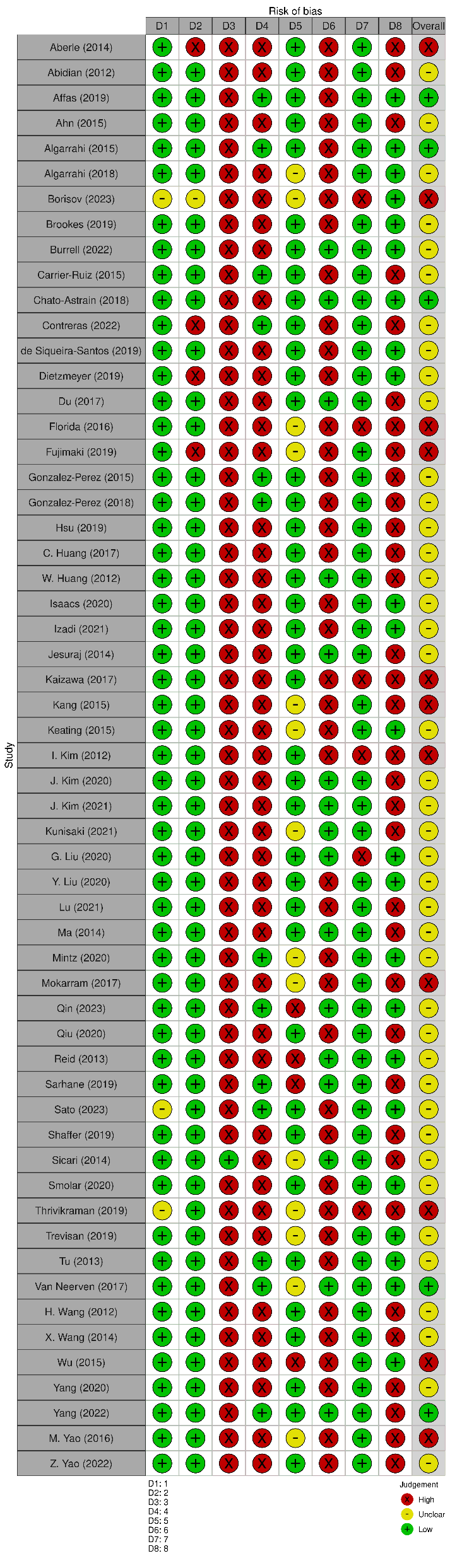

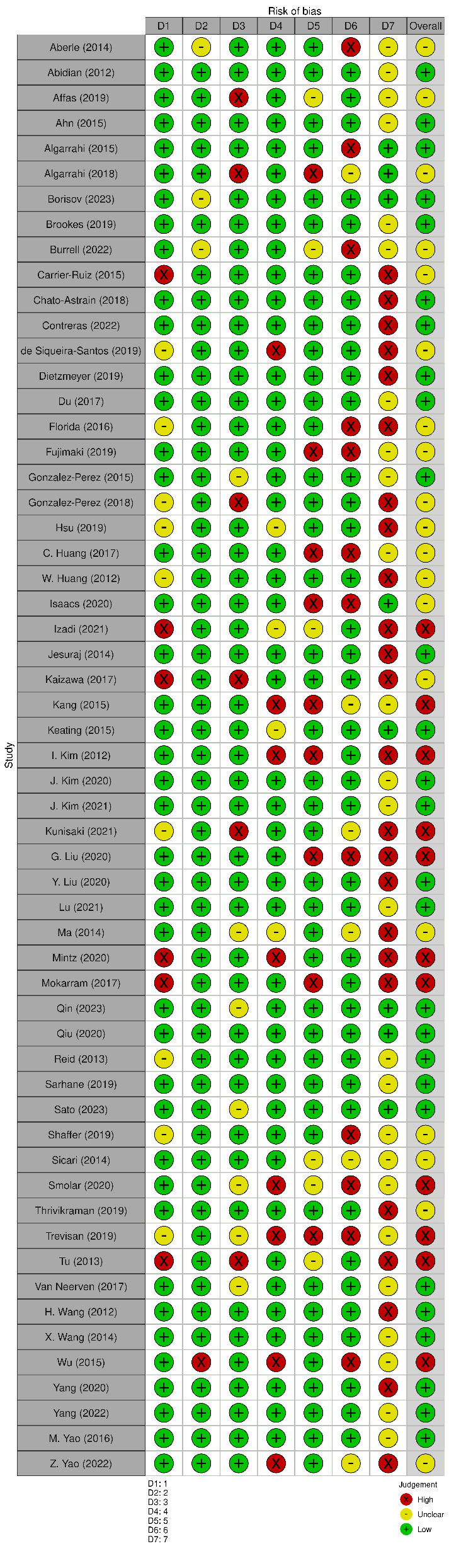


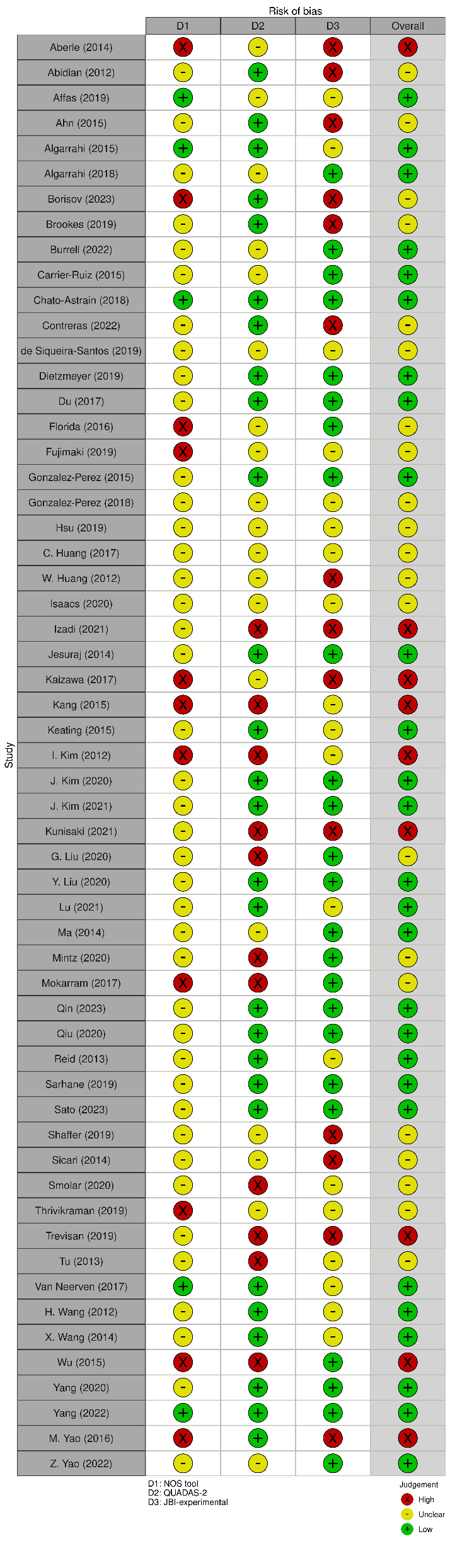

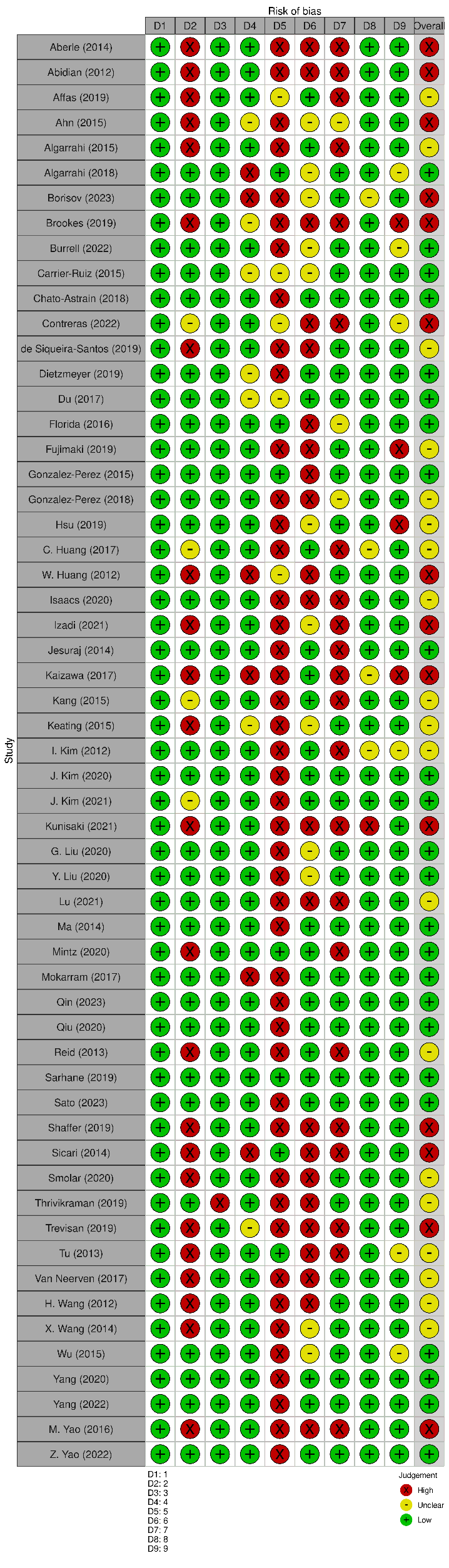


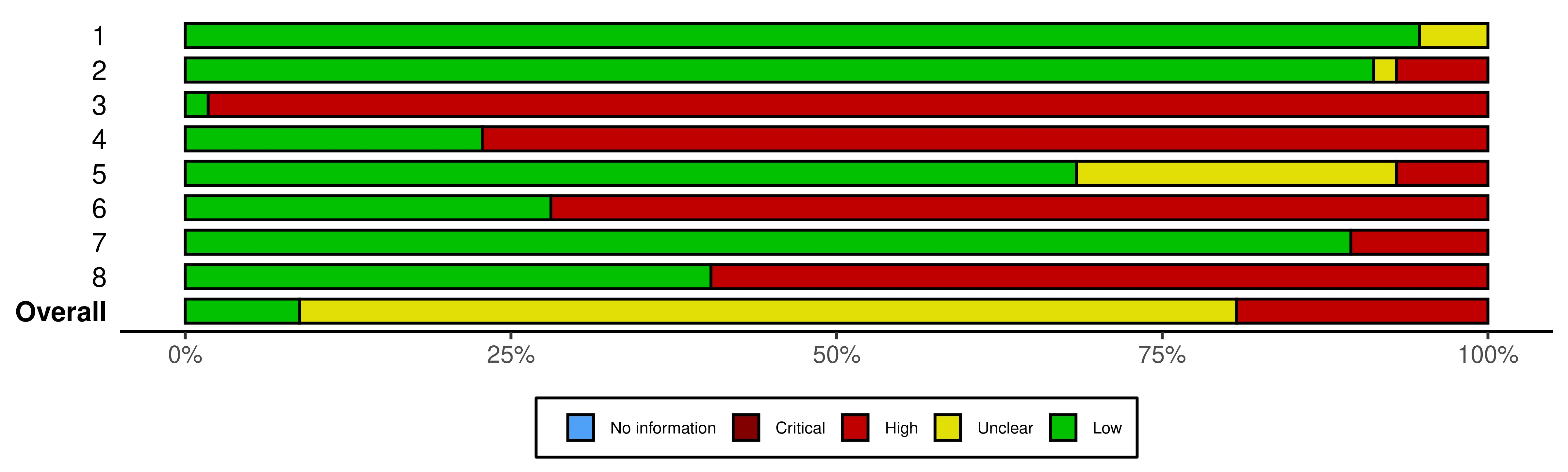


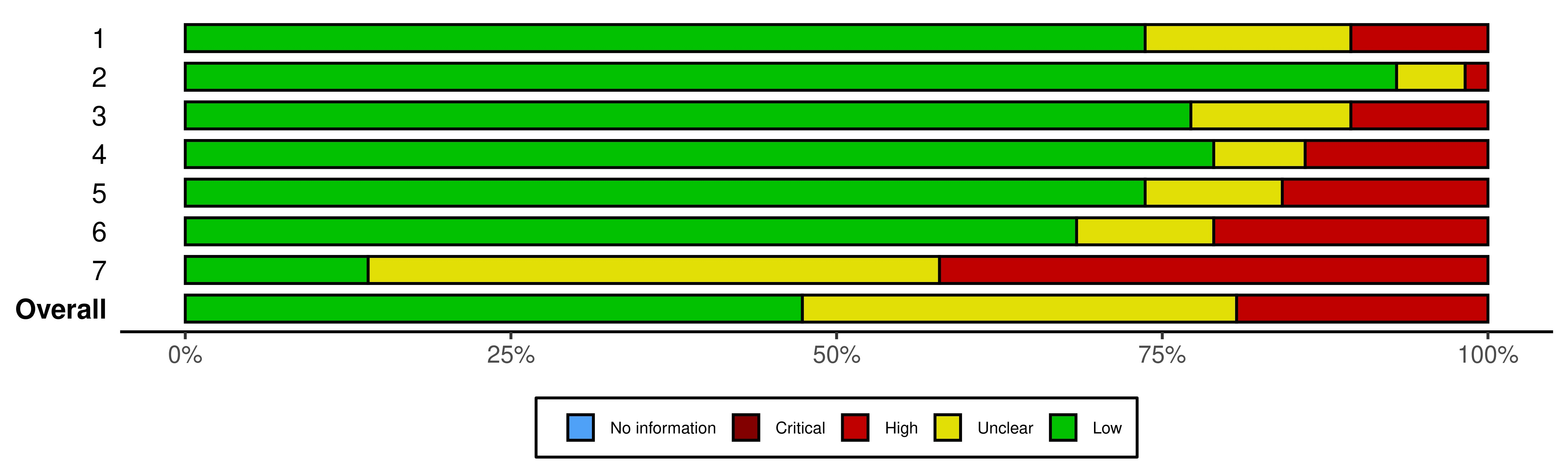


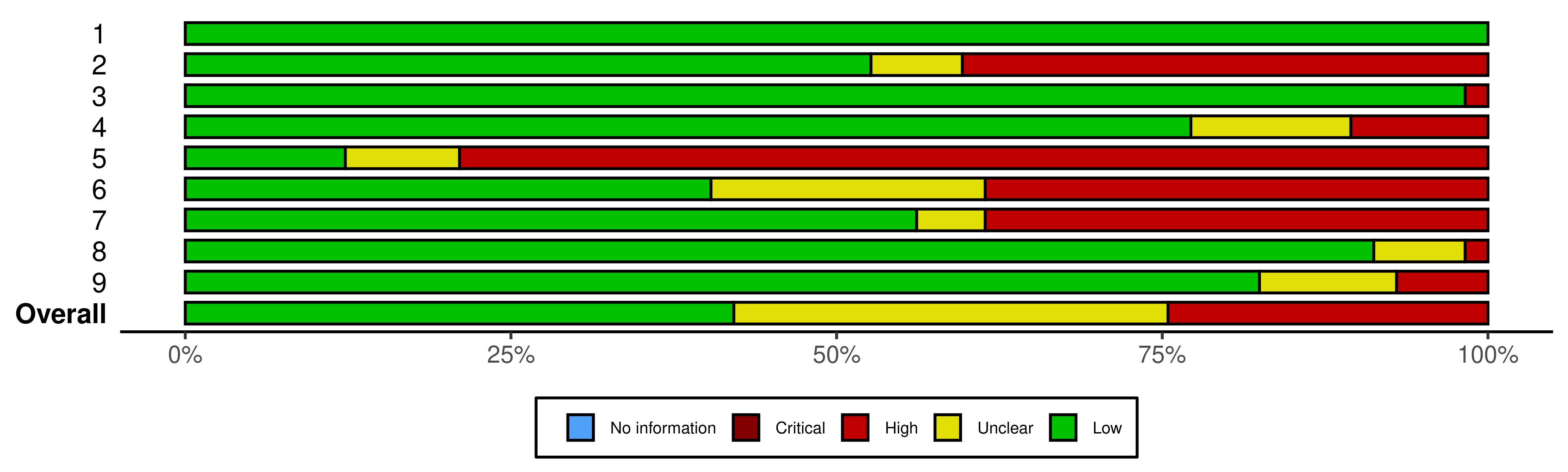


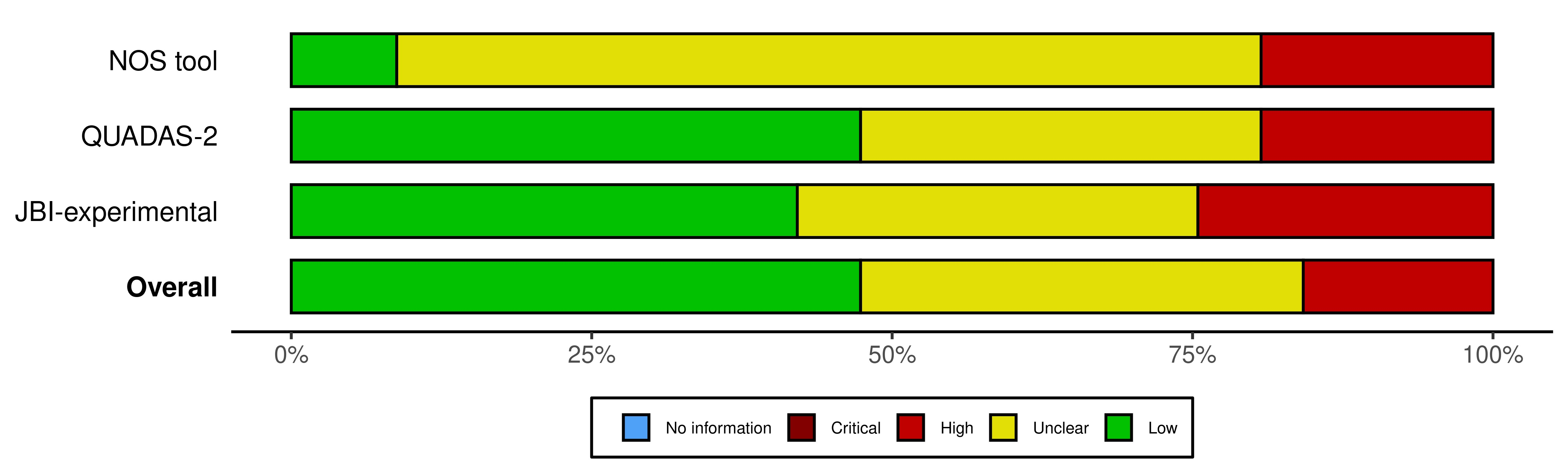


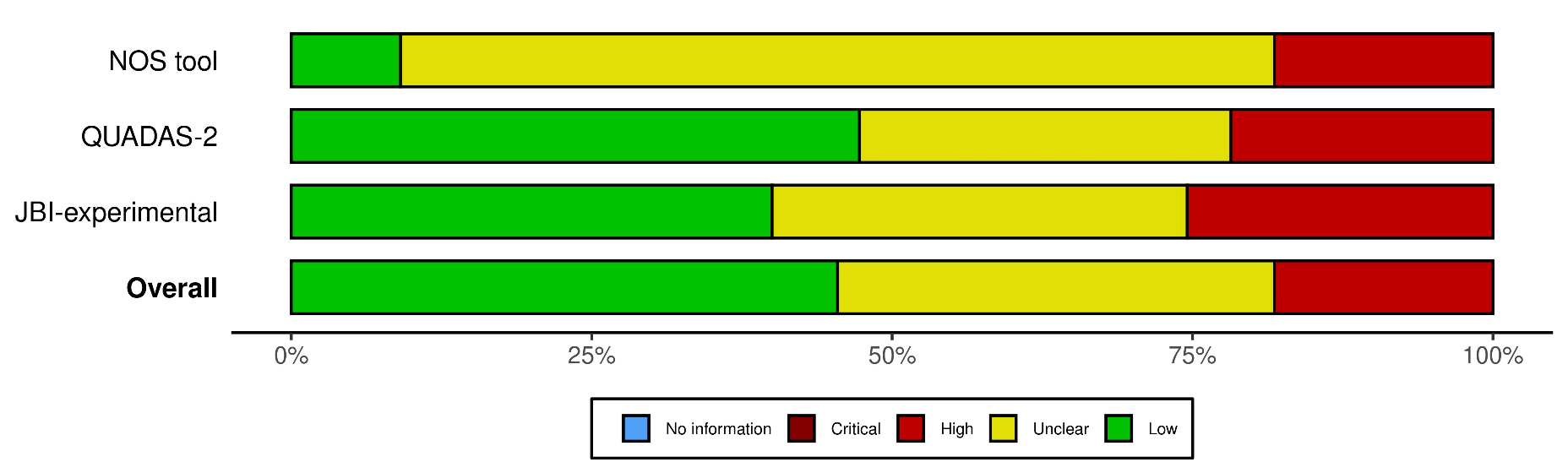

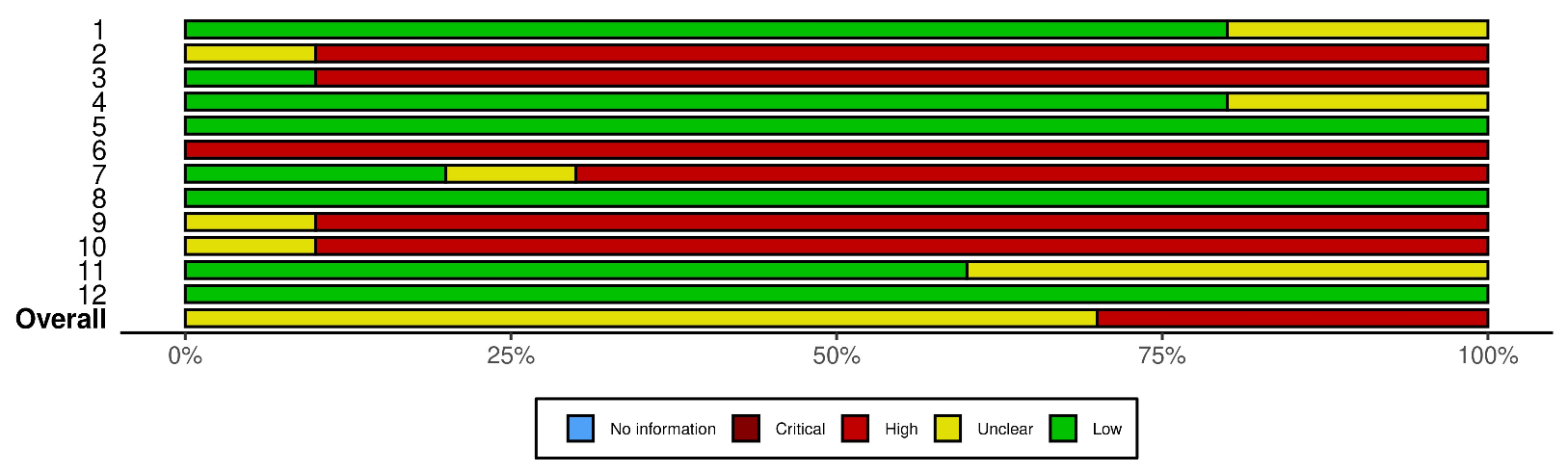

Supplement: sj-docx-3-tej-10.1177_20417314251316918 – Supplemental material for Advances in tissue engineering of peripheral nerve and tissue innervation – a systematic review [file sj-docx-3-tej-10.1177_20417314251316918.docx]
